# Absence in CX3CR1 receptor signaling promotes post‐ischemic stroke cognitive function recovery through suppressed microglial pyroptosis in mice

**DOI:** 10.1111/cns.14551

**Published:** 2024-02-07

**Authors:** Yangyang Ge, Juexi Yang, Jiayi Chen, Maosha Dai, Xiaoke Dou, Shanglong Yao, Chenye Yao, Yun Lin

**Affiliations:** ^1^ Department of Anesthesiology, Union Hospital, Tongji Medical College Huazhong University of Science and Technology Wuhan China; ^2^ Institute of Anesthesia and Critical Care Medicine, Union Hospital, Tongji Medical College Huazhong University of Science and Technology Wuhan China; ^3^ Department of Neurology, Union Hospital, Tongji Medical College Huazhong University of Science and Technology Wuhan China

**Keywords:** cognitive dysfunction, CX3C chemokine receptor 1, ischemic stroke, microglia

## Abstract

**Background:**

Post‐stroke cognitive impairment (PSCI) is a major source of morbidity and mortality after stroke, but the pathological mechanisms remain unclear. Previous studies have demonstrated that the CX3CR1 receptor plays a crucial role in maintaining an early protective microenvironment after stroke, but whether it persistently influences cognitive dysfunction in the chronic phase requires further investigation.

**Methods:**

Mouse was used to establish a middle cerebral artery occlusion (MCAO)/reperfusion model to study PSCI. Cognitive function was assessed by the Morris water maze (MWM) and the novel object recognition test. Neurogenesis was assessed by immunofluorescence staining with Nestin^+^/Ki67^+^ and DCX^+^/BrdU^+^ double‐positive cells. The cerebral damage was monitored by [^18^F]‐DPA‐714 positron emission tomography, Nissel, and TTC staining. The pyroptosis was histologically, biochemically, and electron microscopically examined.

**Results:**

Upon MCAO, at 28 to 35 days, CX3CR1 knockout (CX3CR1^−/−^) mice had better cognitive behavioral performance both in MWM and novel object recognition test than their CX3CR1^+/−^ counterparts. Upon MCAO, at 7 days, CX3CR1^−/−^ mice increased the numbers of Nestin^+^/Ki67^+^ and DCX^+^/BrdU^+^ cells, and meanwhile it decreased the protein expression of GSDMD, NLRP3 inflammasome subunit, caspase‐1, mature IL‐1β/IL‐18, and p‐P65 in the hippocampus as compared with CX3CR1^+/−^ mice. In addition, CX3CR1^−/−^ mice could reverse infarct volume in the hippocampus region post‐stroke.

**Conclusion:**

Our study demonstrated that CX3CR1 gene deletion was beneficial to PSCI recovery. The mechanism might lie in inhibited pyroptosis and enhanced neurogenesis. CX3CR1 receptor may serve as a therapeutic target for improving the PSCI.

## Graphical abstract

Mice deficient in CX3CR1 receptor show improved post‐stroke cognitive impairment (PSCI). Mice deficient in the CX3CR1 receptor have inhibited the microglia pyroptosis‐induced neuroinflammation following ischemic stroke.

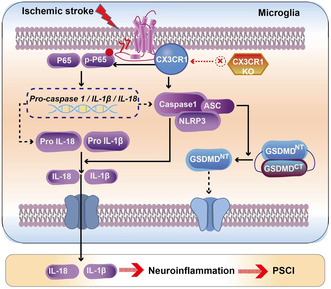

## 1 INTRODUCTION

Stroke accounts for almost 5% of all disability‐adjusted life‐years and 10% of all deaths worldwide.^1^ Ischemic stroke represents the majority of all strokes globally^2^ and can lead to serious neuropsychiatric sequelae.^3^ In particular, cognitive impairment may inflict more than one‐third of stroke survivors,^4^ but its complex molecular mechanism remains poorly understood. Therefore, it is important to identify the key pathological factors to ameliorate post‐stroke cognitive impairment (PSCI).

Cognitive dysfunction is considered one of the main complications of the chronic phase of ischemic stroke, and it takes at least 6 months to be diagnosed with PSCI.^5^ Several studies have revealed a possible correlation between inflammatory factors and the development of PSCI, such as erythrocyte sedimentation rate (ESR), c‐reactive protein, interleukin‐6 (IL‐6), and IL‐12.^6^, ^7^ Whether inhibiting inflammatory responses in the early period of stroke can also prevent the development of PSCI needs to be further studied.

Over the past decade, it is generally believed that ischemic stroke can induce a series of complexly inflammatory responses in the brain.^8^ Furthermore, the neuroinflammation exerts a detrimental effect on the stroke patients' long‐term neurological outcome.^9^ Microglia are key immune cells that respond to neuroinflammation.^10^ After a stroke, microglia can be activated in a matter of minutes and then undergo morphological changes and secrete inflammatory associate cytokines.^10^, ^11^ In recent years, studies have identified some vital cell cross‐talk signaling pathways along the CX3CL1‐CX3CR1 axis, which mediates neuron–microglia communication under physiological and pathological states.^12^ CX3CL1, an exclusive chemokine ligand secreted from neurons, binds to CX3CR1 receptors to maintain microglia activation.^12^, ^13^ Multiple studies have demonstrated that CX3CR1 knockout mice (CX3CR1^−/−^ mice) had inhibited microglial cytotoxicity,^14^ impaired cognitive function,^15^ and reduced adult hippocampal neurogenesis (AHN),^16^, ^17^ which are usually found in aging and neurodegeneration. However, research with CX3CR1^−/−^ mice mainly focused on inhibiting microglial neurotoxicity and maintaining an early protective microenvironment after stroke.^18^, ^19^ The studies on the role of CX3CR1 receptor signaling pathways in the development and progression of PSCI are scanty.

Previous studies found that CX3CR1^−/−^ mice could significantly reduce the release of the inflammatory cytokine IL‐1β, which has been proven to be involved in the development of PSCI.^19^, ^20^ The secretion of IL‐1β into the extracellular environment is mainly controlled by pyroptosis through N‐terminal cleavage of GSDMD.^21^, ^22^ The NLR family pyrin domain‐containing 3 (NLRP3) inflammasome is the most‐studied type that is activated by ischemic stroke.^23^ Moreover, inhibiting the NLRP3 inflammasome activation was also found to improve cognitive functions in the chronic phase of stroke.^24^, ^25^, ^26^ In this study, we explored whether CX3CR1^−/−^ mice can mediate the NLRP3 inflammasome‐induced microglial pyroptosis after ischemic stroke.

We hypothesized that the CX3CR1 gene deletion could alleviate cognitive dysfunction after ischemic stroke. To verify the assumption, we established a 60‐min middle cerebral artery occlusion (MCAO)/reperfusion mouse model and then assessed the cognitive functions of mice by the Morris water maze (MWM) and the novel object recognition behavioral test. Then, to investigate the potential molecular mechanisms of the CX3CR1 receptor attributed to PSCI onset, we evaluated the process of AHN and the activation of microglial pyroptosis.

## 2 METHODS

### 2.1 Animals

CX3CR1 transgenic adult mice were procured from Jackson Laboratories (Bar Harbor, ME, USA). CX3CR1 gene deletion was generated by replacing the second exon of the CX3CR1 gene with the enhanced green fluorescent protein (GFP) reporter gene and backcrossed for more than 10 generations to C57BL/6 mice.^27^ Our experimental animals were housed in a specific pathogen‐free (SPF) environment with food and water available *ad libidum* and under a 12‐h light–dark cycle during the study period. The temperature and relative humidity were maintained at 23 ± 2°C and 50% ± 10%, respectively. All mice were acclimated to the housing condition for 7 days prior to the experiment. The operation was implemented as per the National Institutes of Health Guidelines for the Care and Use of Laboratory Animals (NIH Publication No. 8023, revised 1978). All the protocols concerning animal handling were approved by the Animal Ethics Committee of Tongji Medical College, Wuhan, China (Permission number: S2419).

### 2.2 Middle cerebral artery occlusion (MCAO)/reperfusion model

Experiments were conducted in adult male CX3CR1^−/−^ mice and CX3CR1^+/−^ mice (aged 8–12 weeks and weighing 24–26 g). According to a previous experimental method,^28^ we established a 60‐min MCAO/reperfusion model. First, animals were anesthetized with 2% isoflurane in O_2_. The right common carotid artery, external carotid artery (ECA), and internal carotid artery (ICA) were sequentially isolated by using microsurgical instruments. Next, a customized wire plug of the silicone head was passed through the right ECA into the ICA to occlude the blood supply of the middle cerebral artery (MCA). Finally, after 60 min of cerebral ischemia, the wire plug was carefully removed to restore the blood flow for reperfusion. Then, the vessel was ligated and the wound sutured. Meanwhile, the same operations were performed in both the sham‐operation (sham group) and the surgery groups, except that, in the sham‐operation group, no wire plugs were placed into the MCA. The body temperature of the mice was maintained at 37 ± 0.5°C during the procedure until the mice recovered from the operation.

### 2.3 TTC staining and Nissl staining

The infarct size of the brain was measured by 2,3,5‐triphenyl tetrazolium chloride (TTC) staining. The brain tissue samples were minced into 2‐mm‐thick slices 3 days after the establishment of MCAO. The brain slices were incubated in a 37°C incubator for 10 min. Then, 4% paraformaldehyde was used to fix the tissue for 6 h. Each single slice was photographed, and the infarct size was assessed using the Image J software package by using a previously reported method.^29^ Nissl staining was performed to determine the volume of brain atrophy. Brain sections were incubated with 1% toluidine blue solution at 60°C for 40 min. The volume of brain atrophy was measured by utilizing Image J software.^29^

### 2.4 Behavioral tests

To assess whether CX3CR1 gene deletion promotes neuron recovery after ischemic stroke, an array of behavioral tests was conducted following MCAO, and the investigators were blinded to the experimental grouping.

1. Modified neurological severity scoring (mNSS): The mNSS assessment consists of motor, reflex, and balance tests and can dynamically evaluate the neurological deficits on the day(s) 0, 1, 3, 5, 7, 14, 28, and 35 after reperfusion. The mNSS scores range from 0 to 14 points, and the higher the score, the more severe the neurological impairment (Table 1).^30^
2. Corner test: Mice were placed between two plates (simulated corner) at an angle of 30° and their turning behavior, either to the left or right after entering a corner, was recorded 10 times for each mouse. The turning behavior of mice in the sham group after entering the corner was random. However, the turning of mice in the MCAO group after entering the corner tended to toward the non‐injured side brain.
3. MWM test: The MWM test was performed to evaluate spatial learning and memory in a blinded manner. The maze equipment consisted of a circular tank with a diameter of 120 cm and a platform with a diameter of 6 cm. The water level was 1 cm above the platform, and the water was in an opaque white state, with the temperature maintained at 20 ± 1°C. The entire experiment was video‐recorded and analyzed by matching software [WMT‐100] provided by TaiMeng Technology Co., Ltd. (Chengdu, China). The experiment mainly consisted of two stages: the learning stage was divided into 5 days, with each day having three training sessions. During each session, a mouse on training had to learn to find the hidden platform within 90 s. If the mouse failed to do so within the specified time, it was guided to the platform and then kept there for 15 s. After the learning stage, the mice were allowed to rest for 1 day. In the memory experiment, the hidden platform was removed. Each mouse was placed in the maze, alone, and was left to swim for 60 s. The speed and percentage of time spent in the quadrant where the platform was located were analyzed. A previously reported protocol was used for the experiment.^31^
4. The novel object recognition test: First, the mouse was placed in a rectangular arena and exposed to two identical objects (two red cylinders) for 10 min. Then, the mouse was exposed to a familiar object (one of the foregoing red cylinders) and a novel object (a blue cone) for 10 min after 1 h. The time spent on exploring the familiar and novel object was recorded to calculate the discrimination index (DI). DI = (time spent in novel object − time spent in familiar object)/(time spent in novel object + time spent in familiar object).

**TABLE 1:** Modified neurological severity score (mNSS).

Motor tests		Points
Raising the mouse by the tail		3
Flexion of forelimb	1	
Flexion of hindlimb	1	
Head moved more than 10° to the vertical axis within 30 s	1	
Walking on the floor (normal=0; maximum=3)		3
Normal walk	0	
Inability to walk straight	1	
Circling toward the paretic side	2	
Falling down to the paretic side	3	
Beam balance tests (normal=0; maximum=6)		6
Balances with steady posture	0	
Grasps side of beam	1	
Hugs the beam and one limb falls down from the beam	2	
Hugs the beam and two limb fall down from the beam, or spins on the beam (>30 s)	3	
Attempts to balance on the beam but falls off (>20 s)	4	
Attempts to balance on the beam but falls off (>10 s)	5	
Falls off: No attempt to balance or hang on to the beam (<10 s)	6	
Reflexes absence		2
Pinna reflex (a head shake when touching the auditory meatus)	1	
Corneal reflex (an eye blink when lightly when lightly touching the cornea with cotton)	1	
Maximum points		14

*Note*: The mNSS assessment, which consists of motor, reflex, and balance tests, continuously evaluates the neurological deficits on days 0, 1, 3, 5, 7, 14, 28, and 35 after reperfusion. The mNSS scores range from 0 to 14 points, and higher scores indicate more neurological severe impairment.

### 2.5 Electron microscopy

Brain tissues (1 × 1 × 1 mm) dissected from the hippocampal region were successively fixed in 2.5% glutaraldehyde and 1% osmium tetroxide. Then, tissues were dehydrated in a gradient series of ethanol (30%, 70%, 90%, 100%) and embedded in araldite. Afterward, the tissues were cut into 20–60 nm sections. Finally, the thin sections were stained with 3% uranyl acetate and lead citrate and were then scanned by using an H700 transmission electron microscope (Hitachi, Japan).

### 2.6 BrdU labeling and immunofluorescence staining

Cell proliferation of the hippocampal region was assessed by BrdU labeling. Briefly, 6 days after reperfusion, mice were injected intraperitoneally with BrdU three times, with two injections being 8 h apart. BrdU was dissolved at 10 mg/mL in saline and 50 mg/kg per injection; Sigma, St. Louis, MO, USA. Then, the mice were sacrificed for immunofluorescence staining 7 days after reperfusion.

Mice were transcardially perfused with 4% paraformaldehyde, and the brain was paraffin‐embedded to avoid green GFP fluorescence of CX3CR1 itself. Brain tissues were cut into 4‐μm‐thick coronal sections, and immunofluorescence stained as previously described.^28^ For sections, non‐specific binding was blocked using normal goat serum. Immunoassay was performed using the following antibodies at concentrations (and using protocols) recommended by the respective manufacturers: anti‐GFP (1:400, Abcam), Iba1 (1:200, Cell signaling Technology), anti‐GFAP (1:200, Cell signaling Technology), anti‐Nestin (1:200, Abcam), anti‐Ki67 (1:200, Cell signaling Technology), anti‐GSDMD (1:200, Abcam), anti‐DCX (1:200, Cell signaling Technology), and anti‐BrdU (1:200, Cell signaling Technology). The samples were incubated overnight with primary antibodies at 4°C, and then with appropriate daylight 488‐conjugated goat anti‐mouse (1:400, Abcam) or daylight 549‐conjugated goat anti‐rabbit (1:400, Abcam) secondary antibody in a 37°C incubator for 2 h. All the corresponding secondary antibodies were purchased from Abcam Antibody Company. DAPI (1:1000, Invitrogen, USA) was used for nuclear staining. Images were acquired from five random slides of each brain, with each slide containing eight fields of view within the hippocampal regions. Fluorescent sections were observed under a confocal microscope (Nikon, Japan) and then analyzed by Image J software.

### 2.7 Estimation of microglia number and soma area

Images were captured by using an Olympus fluorescence microscope, at 10× and 40× magnifications. In each slide, the number of GFP‐labeled microglia and the soma area was automatically measured in a given area exclusively containing the entire hippocampal dentate gyrus by using ImageJ software. The measurement was conducted by an experimenter who was blind to the animal grouping. For each brain, microglia number and morphology were assessed in 16 sections along the rostro‐caudal axis of the dorsal hippocampus (in both hemispheres), and the number of microglia and their soma area were averaged.

### 2.8 Western blot assay

The immunoblotting was performed according to our previously established protocol.^32^ Tissue samples were lysed in RIPA buffer containing protease and phosphatase inhibitors (KeyGen Biotech Co., Ltd, Nanjing, China). Then, the protein content of the sample was determined by employing a BCA assay kit. The same amount of sample protein was subjected to SDS–PAGE, transferred onto PVDF membranes, and probed with primary antibodies against CX3CR1 (1:1000, Abcam), the Mouse Reactive Inflammasome Antibody Sampler Kit (1:1000, Cell signaling Technology), gasdermin D (1:1000, Abcam), IL‐18 (1:1000, Abcam), and β‐actin (1:3000, ABclonal, China). The samples were incubated overnight with primary antibodies at 4°C. After washing, the membranes were treated with horseradish peroxidase (HRP)‐conjugated secondary antibody (1:3000, Cell signaling Technology) by incubation for 2 h at room temperature. Enhanced chemiluminescence (ECL) Western Blotting Detection Reagents (Millipore, Billerica, MA, USA) plus BioWest were utilized to enhance the chemiluminescence (UVP, Upland, CA, USA). Band intensity was quantified with ImageJ software (National Institutes of Health, Bethesda, MD, USA). The target protein expression of the samples was normalized to β‐actin, and the phosphorylated protein expression was compared against total protein. All the original, uncropped images of gels/blots are provided in the Figure S1.

### 2.9 Positron emission tomography (PET)/computed tomography (CT) and data analysis

Before PET, the mice were intraperitoneally injected with approximately 50 ± 10 μCi [^18^F]‐DPA. After allowing for uptake for 30 min of [^18^F]‐DPA, the mice were anesthetized with 2% isoflurane and put on a scanning bed. PET/CT images were obtained in static mode for 10 min, and subjected to a CT scan in normal mode by using the Trans PET Discoverist 180 system (Raycan Technology Co., Ltd., Suzhou, China). The PET images were reconstructed by utilizing the three‐dimensional (3D) OSEM method with a voxel size of 0.5 × 0.5 × 0.5 mm^3^. CT images were reconstructed using the FDK algorithm with a 256 × 256 × 256 matrix. Images were displayed with AMIDE (Amide's Medical Imaging Data Examiner) and Pmod (Pmod Technologies LLC, Switzerland) software. The mean standardized uptake value (SUV) was calculated by employing the following formula: Mean pixel value with the decay‐corrected region‐of‐interest activity (μCi/kg)/(Injected dose [μCi]/weight [kg]).^33^

### 2.10 Enzyme‐linked immunosorbent assay (ELISA)

The expression of cytokines IL‐1β and IL‐18 was detected by using mouse IL‐1β and IL‐18 ELISA kits (Neobioscience Technology Co, Ltd, China). The experimental protocols followed the manufacturer's instructions. The absorbance of the samples was measured on a microplate reader (Thermo Fisher Scientific, USA), while the cytokines IL‐1β and IL‐18 concentrations were quantitatively analyzed by generating a standard curve.

### 2.11 Statistical analysis

All data were statistically analyzed by using GraphPad Prism (version 8.0, USA) and were expressed as the means ± SEM. First, all data were subject to tests for normality by the Kolmogorov–Smirnov test. Then, we employed the two‐tailed unpaired Student's *t*‐test to evaluate the significance of the difference between the two groups. When more than two groups and only one factor were involved, the one‐way analysis of variance (ANOVA) was used and followed by Tukey's multiple comparisons test. If more than two groups and two factors were involved, data were analyzed by two‐way ANOVA followed by the Bonferroni's or Tukey test post hoc test. Finally, if data do not exhibit a normal/Gaussian distribution we use a non‐parametric equivalent to analyze it. For all the tests, the threshold of statistical significance was set at a *p*‐value<0.05.

## 3 RESULTS

### 3.1 Improved cognitive function in CX3CR1 ^−/−^ mice after ischemic stroke

**FIGURE 1:**
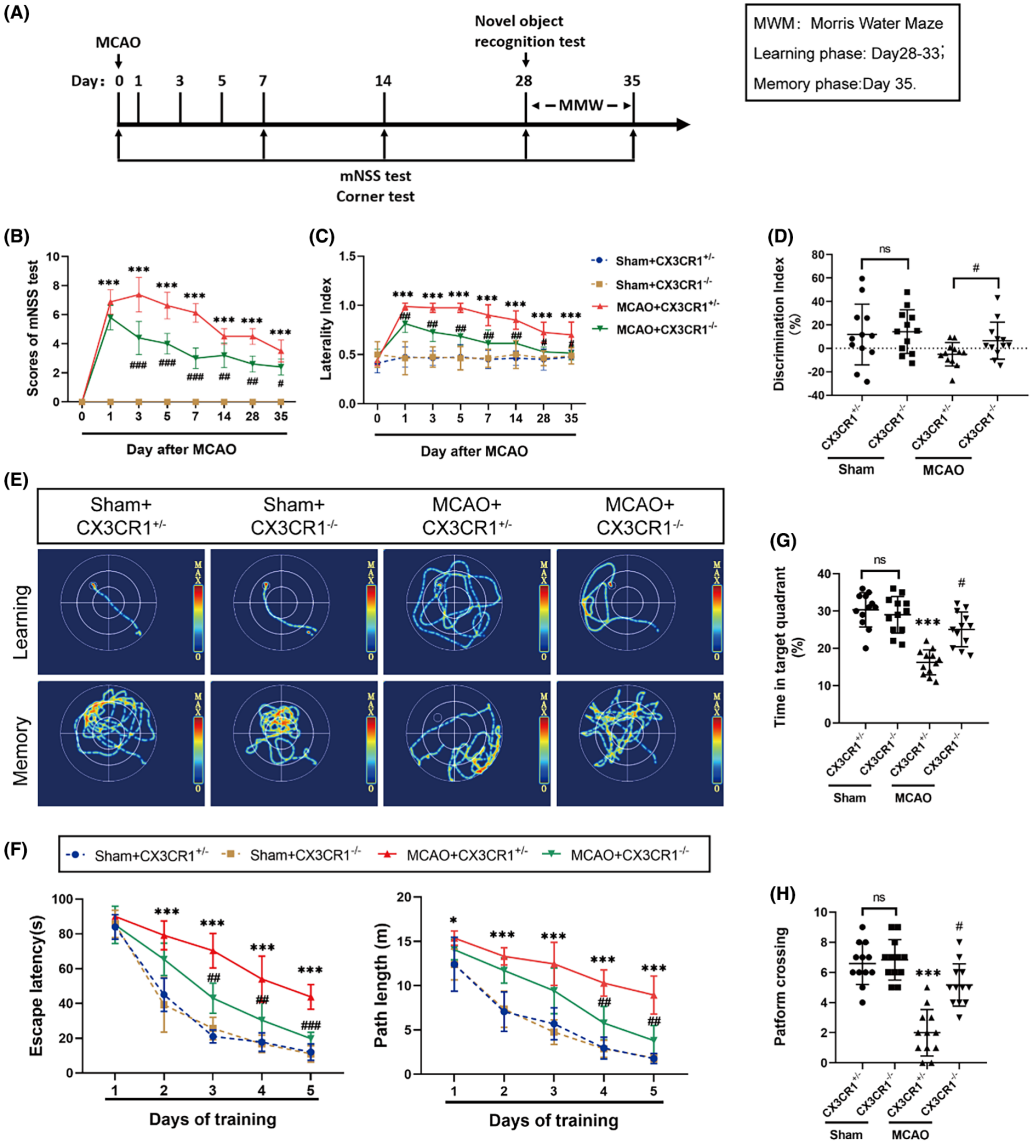
CX3CR1^−/−^ mice enhanced the post‐ischemic stroke cognitive functional recovery. (A) Schematic showing the time of MCAO and behavior tests. CX3CR1 deficiency enhanced sensorimotor function recovery in both the mNSS test (B) and corner test (C). The novel object recognition test (D) and Morris water maze (E‐H) were employed to monitor the cognitive impairment. The behavior assessments were performed at 0, 1, 3, 5, 7, 14, 28, and 35 days after MCAO. *N* = 12–18. All data are shown as the mean ± SEM. *p < 0.05, **p < 0.01, ***p < 0.001 versus Sham+CX3CR1^+/−^ group; ^#^ *p* < 0.05, ^##^ *p* < 0.01, ^###^ *p* < 0.001 versus MCAO+CX3CR1^+/−^ group.

We established a classic 60‐min MCAO/reperfusion mouse model and assessed the cognitive function both in CX3CR1^−/−^ mice and CX3CR1^+/−^ mice. The various behavioral tests are schematically illustrated in Figure 1A.

In the mNSS test, the mice in the MCAO group scored significantly higher than those in the sham groups 1, 3, 5, 7, 14, 28, and 35 day(s) after reperfusion (Figure 1B; *p* < 0.001). After reperfusion, the mNSS scores were significantly lower in CX3CR1^−/−^ mice than in their CX3CR1^+/−^ counterparts (Figure 1B; at 3, 5, and 7 days, *p*<0.001; at 14 and 28 days, *p* < 0.01; at 35 days, *p*<0.05). During the corner test, mice in the MCAO group out‐performed those in the sham group in terms of laterality index (Figure 1C; *p* < 0.001). However, after MCAO, the CX3CR1^−/−^ mice group had a lower laterality index as compared to the CX3CR1^+/−^ group (Figure 1C; at 1, 3, 5, 7, and 14 days, *p* < 0.01; at 28 and 35 days, *p* < 0.05).

In the novel object recognition test, the CX3CR1^+/−^ mice in the MCAO group failed to distinguish between the new and familiar objects, and the discrimination index (DI) was significantly lower in the MCAO group than in the sham group 28 days after MCAO (Figure 1D; *p* < 0.05). However, the DI was significantly higher in the CX3CR1^−/−^ group than in the CX3CR1^+/−^ group after MCAO (Figure 1D; *p* < 0.05). In the sham group, no difference was found between CX3CR1^+/−^ mice and CX3CR1^−/−^ mice (Figure 1D; *p* > 0.05).

In the MWM test, the typical moving trajectory of mice in the MWM test is shown in Figure 1E. Our results exhibited that, after MCAO, the CX3CR1^−/−^ mice covered a shorter distance (Figure 1; *p* < 0.05) and spent less time (Figure 1F; *p* < 0.001) in reaching the hidden platform than CX3CR1^+/−^ mice in the training phase. In the probe phase, the mice that underwent MCAO stayed less time in the target quadrant (Figure 1G; *p* < 0.001) and had a reduced number of platform crossovers (Figure 1H; *p* < 0.001) compared with the sham mice. However, the CX3CR1^−/−^ mice spent more time in the target quadrant (Figure 1G; *p* < 0.001) and the number of platform crossovers was greater as compared with the CX3CR1^+/−^ mice after MCAO (Figure 1H; *p* < 0.001). These findings indicated that our established classic 60‐min MCAO/reperfusion model suffered from significantly impaired cognitive and sensorimotor function in the chronic phase, but the CX3CR1 gene deletion could improve those functions in post‐stroke mice.

### 3.2 Enhanced neurogenesis in the ipsilateral hippocampal regions of CX3CR1 ^−/−^ mice after ischemic stroke

**FIGURE 2:**
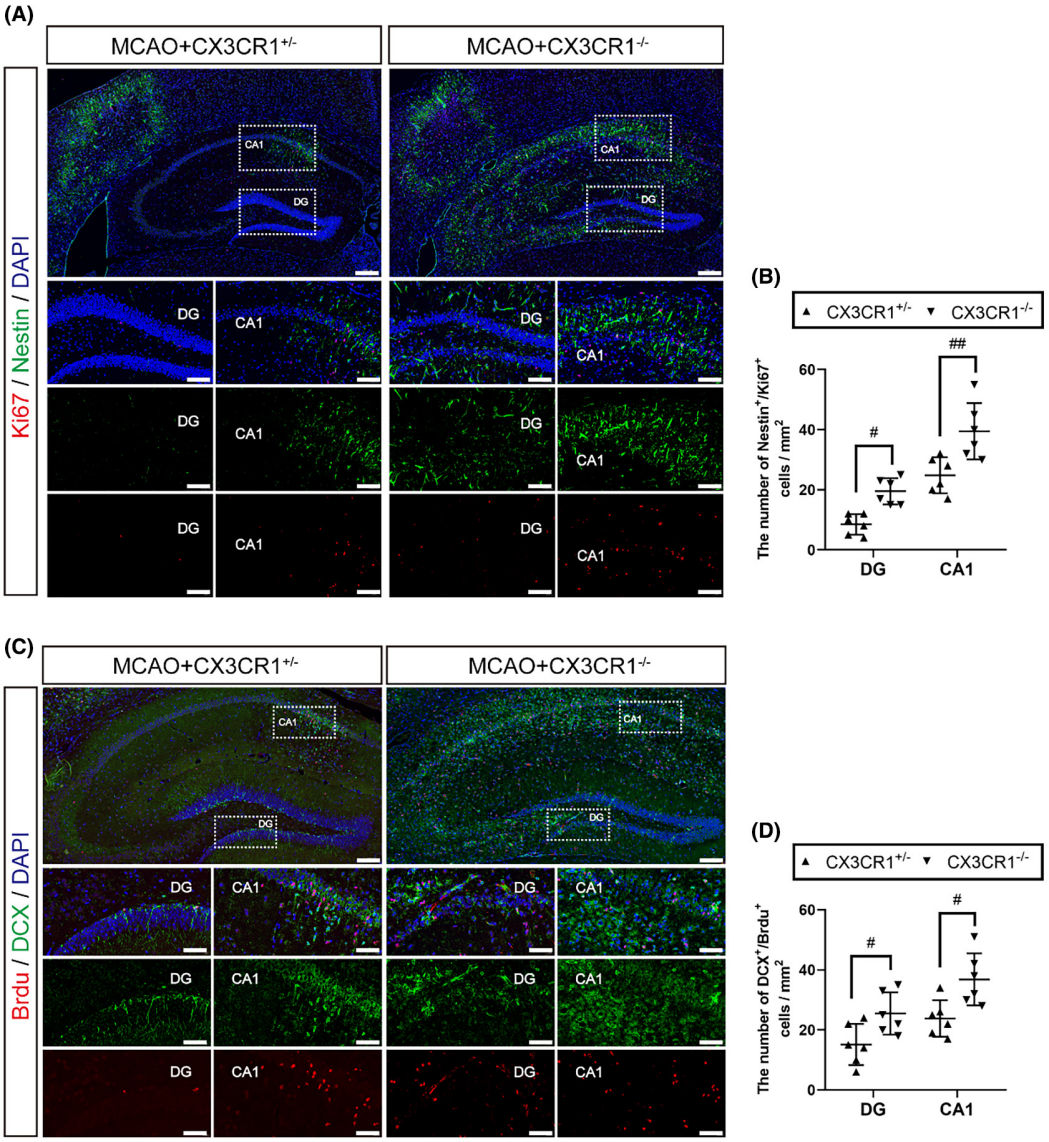
CX3CR1^−/−^ mice promoted adult hippocampal neurogenesis (AHN) after ischemic stroke. (A) Representative images showing the brain sections immunostained with Nestin (green), a specific marker of NSPCs, and the endogenous marker of cell proliferation Ki67 (red) in the ipsilateral hippocampal regions at 7 days after ischemic stroke in mice. (scale bar:200 μm and 50 μm;) (B) The number of Nestin^+^/Ki67^+^ cells in CA1 and DG sub‐regions was quantified and analyzed. (C) Representative images showing the brain sections immunostained with DCX (green) and BrdU (red) in the ipsilateral hippocampal regions at 7 days after ischemic stroke in mice. (scale bar:100 μm and 20 μm) (D) The number of DCX^+^/Brdu^+^ cells in CA1 and DG were quantified and analyzed. *N* = 6. All the data are shown as the mean ± SEM. *p < 0.05, ***p < 0.001 versus Sham+CX3CR1^+/−^ group; ^#^ *p* < 0.05, ^###^ *p* < 0.001 versus MCAO+CX3CR1^+/−^ group.

We further assessed the effect of CX3CR1 gene deletion on the neurogenesis in the ipsilateral hippocampal regions during the first 7 days after MCAO. Representative immunofluorescence images are shown in Figure 2A, and the results indicate that the CX3CR1^−/−^ mice had an increased number of Ki67 and Nestin (a marker of neural stem cells) double‐positive cells in the ipsilateral dentate gyrus (DG) regions (Figure 2B; *p* < 0.05) and CA1 regions (Figure 2B; *p* < 0.001) compared with the CX3CR1^+/−^ mice after MCAO. Meanwhile, in CX3CR1^−/−^ mice, the numbers of DCX (a neuroblast marker) and BrdU double‐positive cells were significantly increased in the ipsilateral DG region (Figure 2C,D; *p* < 0.05) and CA1 region (*p* < 0.05) compared with the CX3CR1^+/−^ mice after MCAO. Collectively, these results indicate that the CX3CR1 gene deletion could enhance the AHN after ischemic stroke.

### 3.3 Morphological alterations and a non‐phagocytic activated state of microglia in CX3CR1 ^−/−^ mice after ischemic stroke

**FIGURE 3:**
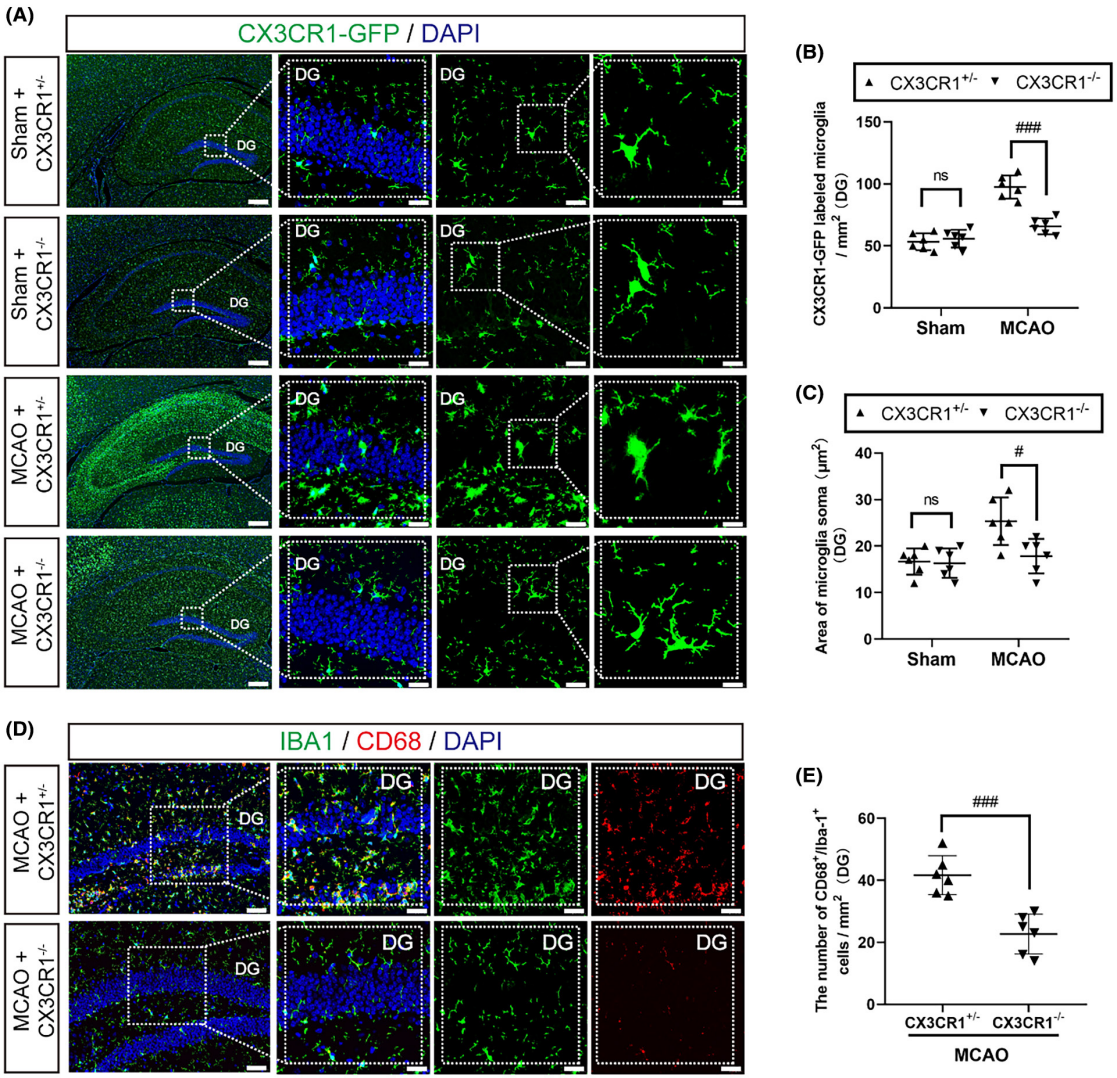
CX3CR1^−/−^ mice showed a non‐phagocytic phenotype of microglia in the dentate gyrus (DG) after ischemic stroke. (A) Representative images and high‐power magnification of the DG immunostained with CX3CR1‐GFP (green) in the ipsilateral brain 7 days after ischemic stroke. (B) Quantification of the number of microglia in the sub‐regions of the DG. (C) Quantification of the area of microglia soma (μm^2^) in the sub‐regions of the DG. (D) Representative images and high‐power magnification of the DG of CX3CR1^+/−^ and CX3CR1^−/−^ mice were showing the expression of microglial activation marker CD68 in Iba‐1^+^ microglia at 7 days after ischemic stroke. (E) Quantification of the expression of CD68^+^/ Iba‐1^+^ microglia in the sub‐regions of the DG. (bar:200 μm, 20 μm, and 10 μm; *N* = 6). All data are shown as the mean ± SEM. *p < 0.05, ***p < 0.001 versus Sham+CX3CR1^+/−^ group; ^#^ *p* < 0.05, ^###^ *p* < 0.001 versus MCAO+CX3CR1^+/−^ group.

To characterize the activation status of microglia in the ipsilateral hippocampal regions 7 days after MCAO, we compared the number of microglia (CX3CR1‐GFP labeling) between CX3CR1−/− mice and CX3CR1+/− mice. Representative immunofluorescence images are given in Figure 3A. The number of CX3CR1‐GFP positive microglia was higher in the MCAO group than in the sham group (Figure 3B; *p* < 0.001), but CX3CR1−/− mice had a lower number of CX3CR1‐GFP positive microglia compared with CX3CR1+/− mice after MCAO (*p* < 0.001). We also found that cerebral ischemic damage could enhance the area of microglia soma in the DG regions, a morphometric indicator of microglial activation. Nonetheless, CX3CR1−/− mice had a smaller area of microglia soma in the DG regions than CX3CR1+/− mice after MCAO (Figure 3A,C; *p* < 0.05). Moreover, we analyzed the phagocytic activity of microglia in terms of the CD68 marker in Iba1+ cells in the ipsilateral hippocampal regions 7 days after MCAO. The expression of CD68+ and Iba1+ double‐positive cells in the hippocampal regions was higher in CX3CR1+/− mice than in their CX3CR1−/− counterparts after MCAO (Figure 3D,E; *p* < 0.001). These observations confirmed that microglia were in a non‐phagocytic state in hippocampal regions in the CX3CR1 gene deletion following ischemic stroke.

### 3.4 Inhibited expression of GSDMD in microglia in CX3CR1 ^−/−^ mice after ischemic stroke

**FIGURE 4:**
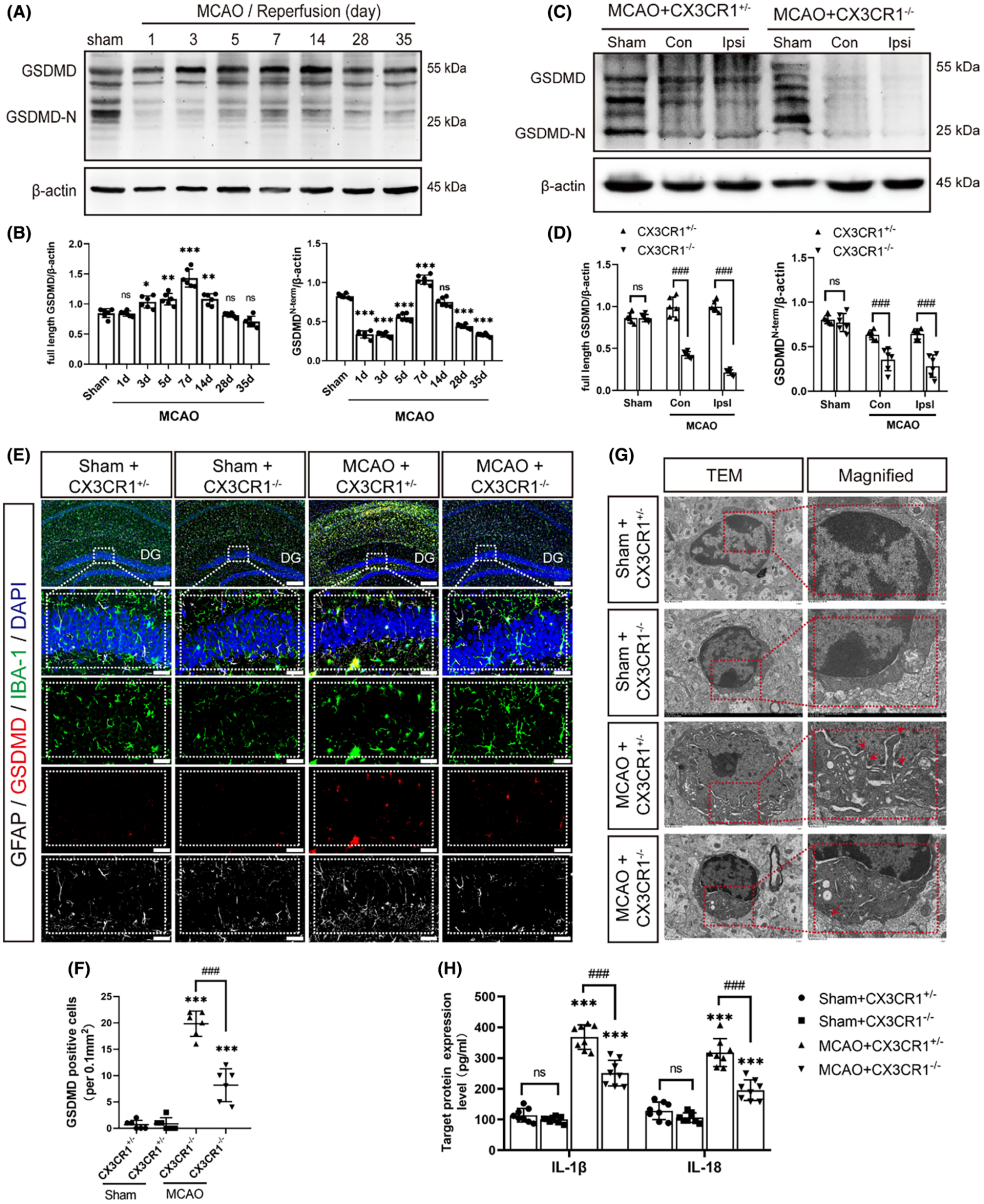
CX3CR1^−/−^ mice suppressed microglial pyroptosis following ischemic stroke. (A and B) Representative immunoblotting imaging and quantitative analysis of GSDMD in the ischemic penumbra at 0, 1, 3, 5, 7, 14, 28, and 35 days after reperfusion, *N* = 6 per group. (C and D) Protein levels of GSDMD in CX3CR1^+/−^ and CX3CR1^−/−^ mice at 7 days after MCAO, *N* = 6 per group. (E and F): Representative immunofluorescence images of GSDMD co‐stain with Iba‐1 and GFAP in the ipsilateral hippocampus region, and quantitative analysis of GSDMD‐positive cells 7 days after reperfusion, *N* = 6 per group. (scale bar:100 μm and 20 μm). (G): Representative transmission electron microscopy images of membrane pores formed on microglia in the ipsilateral hippocampus region at 7 days after reperfusion, *N* = 3 in each group. (scale bar:2.0 μm and 1.0 μm). (H): The ELISA assay kit detected IL‐18 and IL‐1β production in the ipsilateral hippocampus. *N* = 6–8 per group. Data are shown as the mean ± SEM. *p < 0.05, **p < 0.01, ***p < 0.001 versus Sham+CX3CR1^+/−^ group; ^#^ *p* < 0.05, ^##^ *p* < 0.01, ^###^ *p* < 0.001 versus MCAO+CX3CR1^+/−^ group. Con: contralateral hippocampal regions; Ipsi: ipsilateral hippocampal regions.

First, the GSDMD expression was temporally assessed in the hippocampal regions after MCAO. Western blotting showed that the expression of full‐length GSDMD was elevated 3, 5, 7, and 14 days after MCAO as compared with the sham group. but the expression of N‐terminal GSDMD was only elevated at 7 days (Figure 4A,B; *p* < 0.001). We subsequently examined whether CX3CR1 gene deletion could affect the activation of GSDMD protein in the ipsilateral hippocampal regions 7 days after MCAO. Immunoblotting revealed that the CX3CR1^−/−^ mice had significantly reduced levels of full‐length GSDMD (Figure 4C,D; *p* < 0.001) and N‐terminal GSDMD (*p* < 0.05) compared with the CX3CR1^+/−^ mice after MCAO. Moreover, immunostaining exhibited that the GSDMD‐positive cells were mainly co‐stained with Iba1^+^ cells (microglia marker) rather than GFAP^+^ cells (astrocytes marker) in the ipsilateral hippocampal region. The CX3CR1^−/−^ mice also had fewer GSDMD‐positive cells than the CX3CR1^+/−^ mice after MCAO (Figure 4E,F; *p* < 0.001).

TEM showed that membrane pores formed by GSDMD on microglia in the ipsilateral hippocampal region were less in CX3CR1^−/−^ mice than in CX3CR1^+/−^ mice 7 days after reperfusion (Figure 4G). ELISA indicated that the IL‐1β/18 in the ipsilateral hippocampal region was up‐regulated in MCAO group compared with the sham group (Figure 4H; *p* < 0.001). The IL‐1β/18 level was lower in the CX3CR1^−/−^ mice than in CX3CR1^+/−^ mice after MCAO (*p* < 0.001). The aforementioned results suggested that CX3CR1 gene deletion might inhibit the activation of microglial pyroptosis and, at the same time, inhibited the release of IL‐1β/18 in the ipsilateral hippocampal regions after ischemic stroke.

### 3.5 Inhibited NLRP3 inflammasome and P65 activation in CX3CR1 ^−/−^ mice after ischemic stroke

**FIGURE 5:**
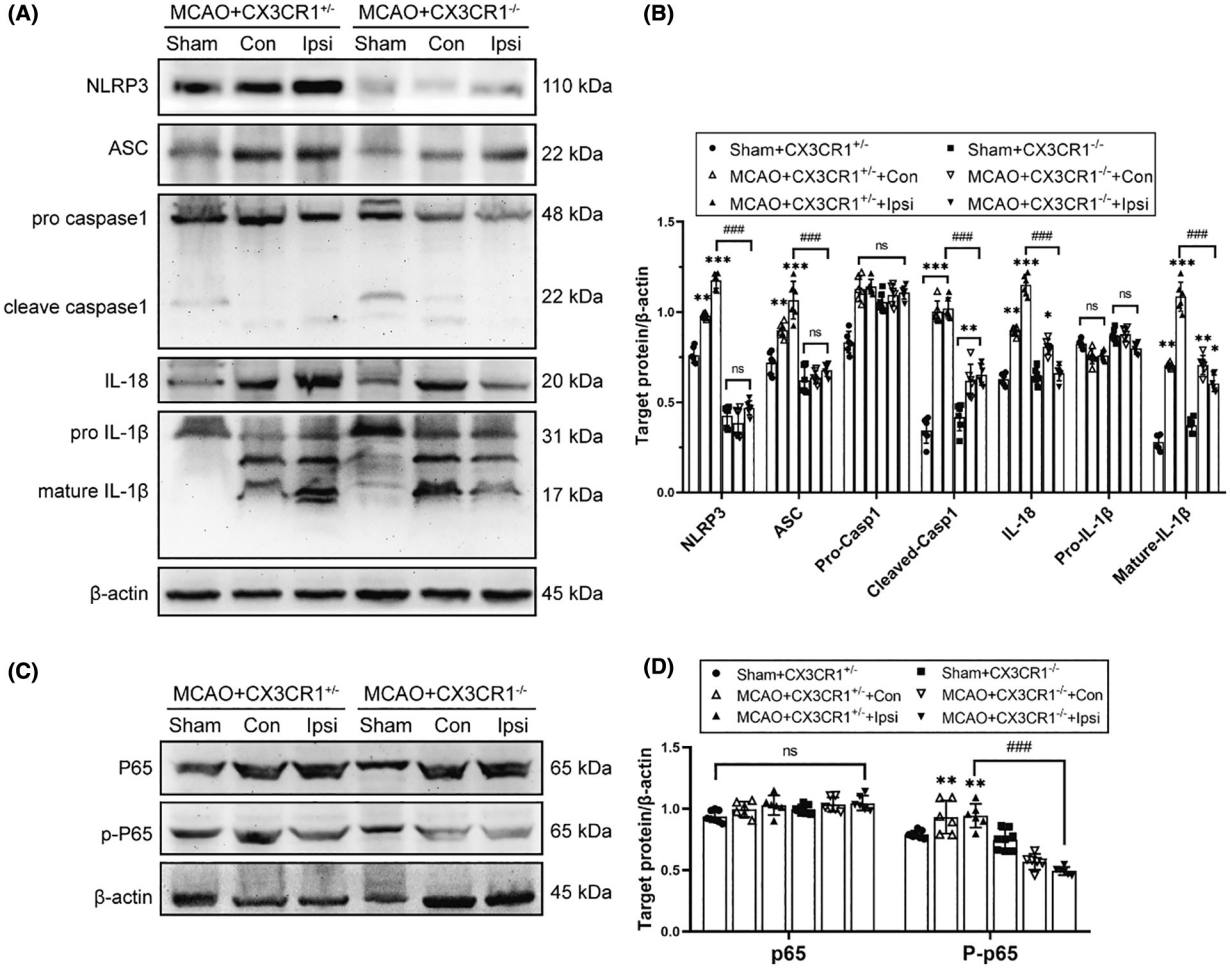
CX3CR1^−/−^ mice inhibited ischemia‐induced NLRP3 inflammasome activation and P65 signaling activation post‐ischemic stroke. (A and B): Representative immunoblotting imaging and quantitative analysis of NLRP, ASC, caspase‐1, IL‐18, and IL‐1β expression in the ipsilateral hippocampus region at 7 days after reperfusion, *N* = 6 per group. (C and D) Immunoblotting and quantitative analysis for p65 and p‐p65 in the ipsilateral hippocampus region 7 days after reperfusion, *N* = 6–8 per group. Data are shown as the mean ± SEM. *p < 0.05, **p < 0.01, ***p < 0.001 versus Sham+CX3CR1^+/−^ group; ^#^ *p* < 0.05, ^##^ *p* < 0.01, ^###^ *p* < 0.001 versus MCAO+CX3CR1^+/−^ group. Con: contralateral hippocampal regions; Ipsi: ipsilateral hippocampal regions.

We further explored whether CX3CR1 gene deletion inhibits NLRP3 inflammasome activation 7 days after MCAO. Our immunoblotting results showed that, in CX3CR1^+/−^ mice after MCAO, NLRP3 inflammasome components (NLRP3, ASC, and cleaved caspase‐1), mature IL‐1β, and IL‐18 were significantly increased as compared with the sham group (Figure 5A,B; *p* < 0.001). However, the enhanced expression of these proteins was decreased in the CX3CR1^−/−^ mice compared with CX3CR1^+/−^ mice after MCAO (*p* < 0.001), but there was no significant difference in pro‐caspase‐1 and pro‐IL‐1β expression (*p* > 0.05).

Furthermore, previous studies indicated that NF–κB signaling is required for NLRP3 inflammasome activation. We next assessed whether CX3CR1 gene deletion could also affect NF–κB signaling activation. Western blotting revealed that the p‐p65 was up‐regulated in CX3CR1^+/−^ mice after MCAO as compared with sham‐operated mice (Figure 5C,D; *p* < 0.01). However, the enhanced expression of p‐p65 protein was significantly inhibited in the CX3CR1^−/−^ mice compared with the CX3CR1^+/−^ mice after MCAO (*p*<0.001). There was no significant difference in p65 protein expression (*p* > 0.05).

### 3.6 Reduced brain infarct volume, cerebral atrophy, and neuroinflammatory reactions in CX3CR1 ^−/−^ mice after ischemic stroke

**FIGURE 6:**
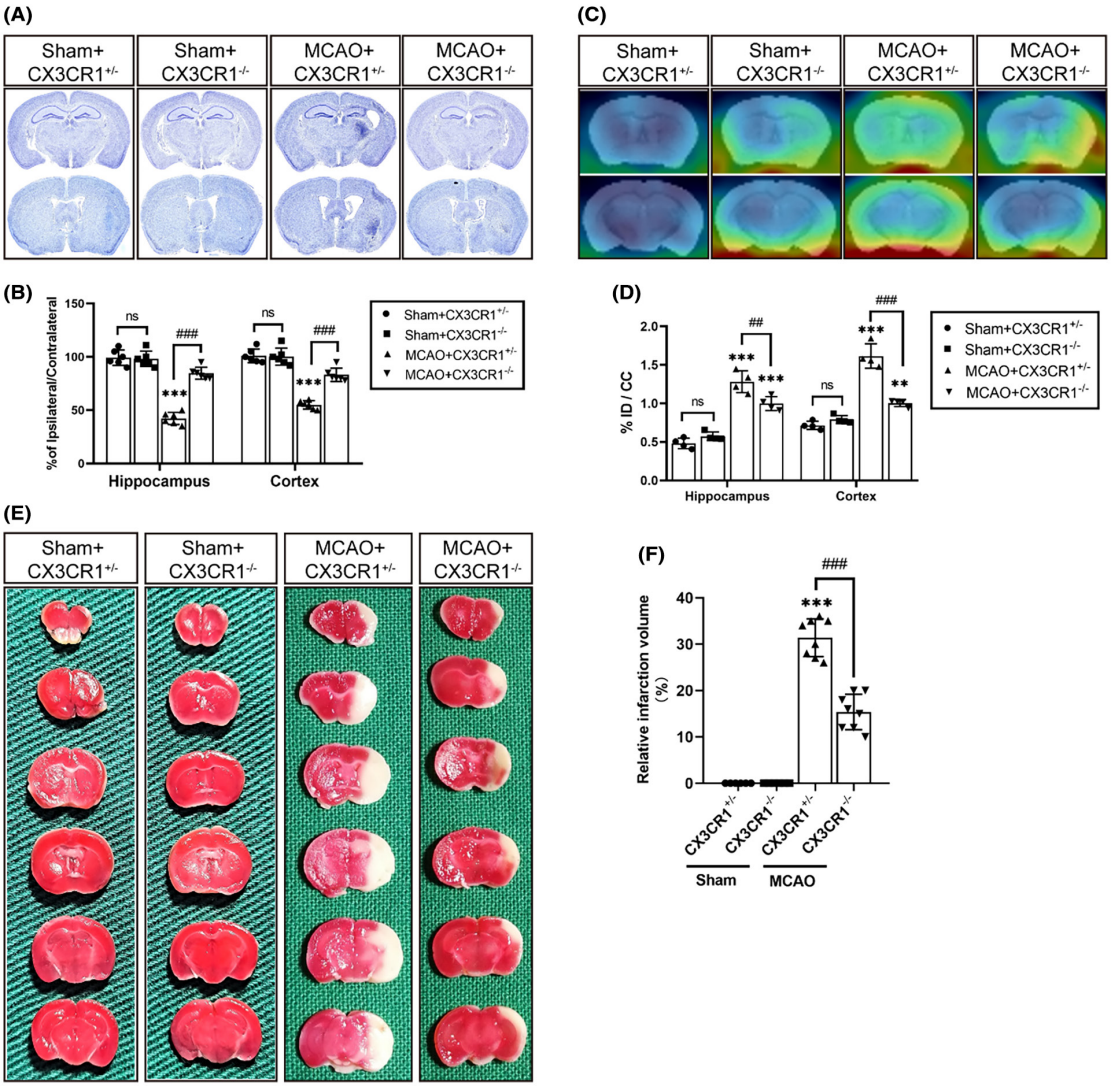
CX3CR1^−/−^ mice reduced brain damage and enhanced the rate of survival post‐ischemic stroke. (A and B) Representative images and quantification of Nissl staining at 7 days after reperfusion. *N* = 6 per group. (C and D) Representative images and quantification of [^18^F] DPA‐714 PET images at 7 days after reperfusion. *N* = 4 per group. (E and F) Representative images and the quantitative analysis of TTC staining at 3 days after reperfusion. *N* = 8 per group. All the data are shown as the mean ± SEM. *p < 0.05, **p < 0.01, ***p < 0.001 versus Sham+CX3CR1^+/−^ group; ^#^ *p* < 0.05, ^##^ *p* < 0.01, ^###^ *p* < 0.001 versus MCAO+CX3CR1^+/−^ group.

We next investigated whether the enhanced cognitive function is accompanied by histological recovery. The infarct volume and the hippocampal volume were determined, respectively, by using TTC staining and Nissl staining. Ischemic stroke mice displayed significantly lower brain volume in the hippocampus than sham mice (Figure 6A,B; *p* < 0.001), indicating that mice with lower hippocampal volume could develop severe cognitive deficits. In addition, in the CX3CR1^−/−^ mice, hippocampal volume was substantially restored as compared to CX3CR1^+/−^ mice after MCAO (Figure 6A,B; *p* < 0.001). Moreover, we used non‐invasive [^18^F]‐DPA‐714 PET imaging to monitor the inflammatory reaction induced by activated microglia in the brain 7 days after reperfusion. The PET imaging showed that in CX3CR1^−/−^ mice, [^18^F]‐DPA‐714 binding was significantly decreased in the ipsilateral hippocampal region as compared with the CX3CR1^+/−^ mice after MCAO (Figure 6C,D; *p* < 0.01). Moreover, the infarct volume in the hippocampus was significantly decreased in CX3CR1^−/−^ mice compared with CX3CR1^+/−^ mice after MCAO (Figure 6E,F; *p* < 0.001). Our results suggested that CX3CR1 gene deletion, reduced ischemic cerebral damage‐induced neuroinflammation in hippocampal regions, may contribute to the improvement of PSCI.

## 4 DISCUSSION

This study demonstrated that ischemic cerebral damage triggered pyroptosis and neurogenesis within 7 days after MCAO, which might participate in the development of PSCI. However, CX3CR1 gene deletion could influence microglia pyroptosis and AHN in the early phase of stroke and then improve cognitive dysfunction in the chronic phase. These results indicated that CX3CR1 signaling might participate in the development of long‐term PSCI.

Stroke‐associated research mostly focused on physical disabilities, while cognitive dysfunction, serious neuropsychiatric sequelae for stroke survivors, has been rather neglected.^4^, ^34^ Clinical data analyses indicated a prevalence of 38% of PSCI in the first year after stroke.^35^ Neuroimaging adds to the prediction of cognitive decline after stroke, in which hippocampal atrophy is a strong predictor for PSCI outcome.^36^ Therefore, we established a mouse model by 60 min MCAO/reperfusion, which caused significant ischemic damage to the hippocampal region. Importantly, as compared with sham groups, in our mouse MCAO model, neurogenesis was significantly reduced in the hippocampus region and the mice exhibited worse cognitive behaviors in the chronic phase. The hippocampus region is implicated in cognitive function in the adult mammal brain.^37^ The DG of the hippocampus region can continuously generate new neurons throughout life, a process known as “adult hippocampal neurogenesis (AHN).”^38^, ^39^ Previous studies demonstrated that AHN was mainly elevated during the 1 to 2 week(s) after MCAO.^38^ AHN can also promote cognitive functional recovery by replenishing lost neurons with newborn neurons after stroke.^39^, ^40^ Our data indicated an inverse correlation between neurogenesis levels and cognitive dysfunction, indirectly supporting the notion that AHN can serve as a modulator of cognitive performance after stroke.

Of note, the CX3CR1 receptor signaling pathway was reported to play an important role in the mediation/modulation of AHN.^41^, ^42^, ^43^ The CX3CL1‐CX3CR1 axis has been proven to could mediate neuron–microglia communication under both physiological and pathological states.^12^ CX3CR1 receptor is expressed exclusively on the surface of microglia in the central nervous system (CNS), and its ligand, CX3CL1, is virtually solely produced by neurons.^44^ Prior studies have reported that CX3CR1^−/−^ mice could experience either impairment^15^ or improvement^41^, ^42^ in cognitive function. For example, CX3CR1^−/−^ mice achieved improved cognitive function by persistently maintaining hippocampal neurogenesis in aging and neurodegenerative diseases.^16^, ^17^ In this study, our results showed that after MCAO, CX3CR1^−/−^ mice had enhanced neurogenesis and improved cognitive function as compared with their CX3CR1^+/−^ counterparts. Our experimental results indicated that the CX3CR1 receptor gene deletion may have an essential regulatory role in promoting the recovery of PSCI.

Previous studies have revealed a possible correlation between inflammatory factors and the development of PSCI.^6^, ^7^ Microglia, the primary residing immune cells in the CNS, acts as an important regulator of neuroinflammation after stroke.^45^ Activated microglia might exert either neuroprotective or detrimental effects on neurons following ischemic stroke.^10^ These dual effects are principally mediated by the CX3CL1/CX3CR1 axis signaling pathway that governs neuron–microglia communication.^12^ Multiple studies found that CX3CL1/CX3CR1 axis signaling mainly mediates the microglial transformation in an “on” or “off” status within physiological or pathological environments.^16^, ^46^ The absence of this physiological “brake” in CX3CR1^−/−^ mice is expected to elicit microglial activation. A study proved that this special type of mice showed dysregulated microglial neurotoxicity in those with neurodegenerative diseases.^14^ However, in a transient MCAO animal model, CX3CR1^−/−^ mice had significantly decreased infarct volume and exhibited neuroprotection associated with the inhibition of the inflammatory milieu, as well as inhibited activation of peripheral macrophages and microglia at the early phase of the ischemic damage‐induced microenvironment.^18^, ^19^, ^20^ In this study, we examined the microglial activation in the ipsilateral hippocampal regions, including the number of Iba1^+^ cells, the size of their nuclei, and the expression of the activation marker CD68. Our observation revealed that, as compared with CX3CR1^+/−^ mice, CX3CR1^−/−^ mice had significantly inhibited microglial phagocytic activation and morphological alterations after stroke.

To further confirm that CX3CR1 gene deletion could inhibit ischemic injury‐induced neuroinflammation, we employed [^18^F]‐DPA‐714 PET imaging to monitor the mouse brain after stroke. Prior studies have reported that the expression of translocator protein (TSPO) was enhanced in activated microglia, and [^18^F]‐DPA‐714 exhibited highly specific binding to TPSO ligands post‐stroke.^33^, ^47^ Our data showed that the CX3CR1^−/−^ mice exhibited a decreased magnitude of the [^18^F]‐DPA‐714 PET imaging signals in the ipsilateral hippocampal region compared with CX3CR1^+/−^ mice after MCAO. Therefore, reducing primary brain injury by CX3CR1 gene deletion to inhibit neuroinflammation early after stroke could improve cognitive function at later time points. We also believe that noninvasive [^18^F]‐DPA‐714 PET imaging can monitor the neuroinflammatory reaction after ischemic stroke.

Neuroinflammation in the peri‐infarct area plays a critical role in PSCI.^29^ Previous studies have also suggested a close relationship between neuroinflammation (particularly indicated by IL‐1 production and microglial activation) and cognitive functions.^41^, ^48^ Our study showed that deletion of the CX3CR1 gene inhibited the secretion of pro‐inflammation cytokines, such as IL‐1β and IL‐18, after MCAO. The mounting interest in inflammasomes has led to attention to their role in neurological diseases, including meningitis, stroke, and Alzheimer's disease.^22^ The NLRP3 inflammasome is the best‐known regulator of pyroptosis activation and can evoke GSDMD‐executed pyroptotic cell death.^23^, ^24^ Our study showed that CX3CR1 gene deletion inhibited GSDMD protein activation after stroke. Furthermore, NF‐κB signaling, serving as a priming signal of the inflammatory response, could trigger an enhanced downstream transcriptional activity of NLRP3 inflammasome in an inflammatory environment.^49^ The NLRP3 inflammasome that binds to ASC and caspase‐1 can mediate the canonical pathway of pyroptosis.^50^ The NLRP3 inflammasome mainly influences the intracellular maturation of the precursors of IL‐1β/18, and its secretion into the extracellular environment triggers neuroinflammation after ischemic stroke.^50^, ^51^ Our study also showed that CX3CR1 gene deletion inhibited the expression of NLRP3, ASC, caspase‐1, IL‐1β, IL‐18, and NF‐κB p65 protein after stroke. In different CNS diseases, inhibition of NLRP3 inflammasome signaling has been reported to regulate neuroinflammation and result in the development of long‐term cognitive impairment, including ischemic stroke [25,26]. Importantly, our study revealed that, after ischemic stroke, microglia pyroptosis was upregulated in the ipsilateral hippocampal region. CX3CR1 gene deletion could significantly suppress microglia pyroptosis and then improve cognitive dysfunction after stroke.

There are still some potential limitations in our study. Such as, the CX3CR1 receptor is also expressed in myeloid origin cells of the peripheral immune system, so future studies will need to further demonstrate the effect of CX3CR1 gene deletion on the role of peripheral myeloid cells following ischemic stroke; Our experimental only found that CX3CR1 gene deletion can increase the number of both DCX+ (a neuroblast marker) and Nestin+ (a marker of neural stem cells) positive neurogenesis cells within the ipsilateral hippocampus after stroke, but whether CX3CR1 gene deletion promotes functional maturation and long‐term survival of these neurogenesis cells requires demonstration by further studies.

## 5 CONCLUSION

In conclusion, this article, for the first time, revealed the correlation between CX3CR1 receptor deletion and improved PSCI. The removal of CX3CR1 receptor signaling significantly inhibited microglial activation and promoted neurogenesis in the hippocampal regions after stroke. Mechanistically, those effects might be ascribed to inhibited microglial pyroptosis induced by NLRP3 inflammasome and diminished release of pro‐inflammation cytokines. Our experimental data showed that PSCI might be treated by targeting CX3CR1 receptor signaling in the brains of patients with ischemic stroke.

## AUTHOR CONTRIBUTIONS

Yangyang Ge wrote the main manuscript text and established the animal models. Juexi Yang performed the experiments. Jiayi Chen collected samples. Maosha Dai and Xiaoke Dou analyzed the data. Chenye Yao and Shanglong Yao revised the manuscript. Yun Lin designed the experiments. All the authors reviewed and approved the final version of the manuscript.

## FUNDING INFORMATION

This work was supported by the National Natural Science Foundation of China (Nos. 81571138 and 82071485).

## CONFLICT OF INTEREST STATEMENT

The authors declare no conflicts of interest.

## CONSENT TO PARTICIPATE

All the experimental protocols were performed in accordance with the National Institutes of Health “Guide for the Care and Use of Laboratory Animals” (NIH Publications No. 8023, revised 2011).

## CONSENT FOR PUBLICATION

Not applicable.

## Supporting information


Figure S1.

## Data Availability

All data generated or analyzed during this study are included in this published article.
