# Supplementary material for: Absence in CX3CR1 receptor signaling promotes post‐ischemic stroke cognitive function recovery through suppressed microglial pyroptosis in mice
Source: CNS Neurosci Ther. 2024 Feb 7;30(2):e14551. doi: 10.1111/cns.14551 (PMC10850801; doi:10.1111/cns.14551)
Supplement: Supplementary file 1 — Figure S1. [file CNS-30-e14551-s001.zip › Supplement Figure.docx]

**Supplement Figure：**The original, uncropped image of gels/blots appearing in the manuscript.

Supplement Figure 1A: Full unedited gel/blot for Figure 4A (GSDMD/GSDMD-N)


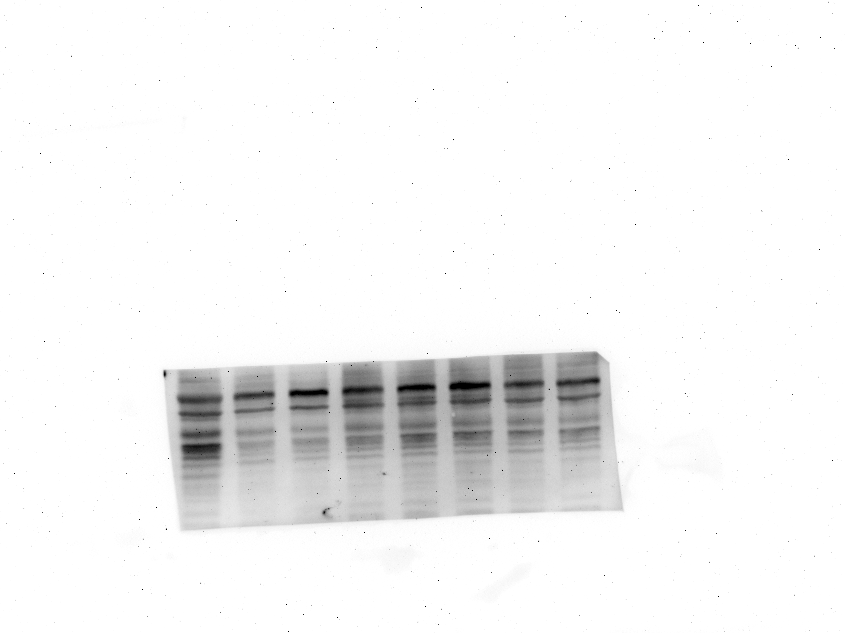


**55kDa**

**25kDa**

**GSDMD**

**GSDMD-N**

Supplement Figure 1B: Full unedited gel/blot for Figure 4A (β-actin)


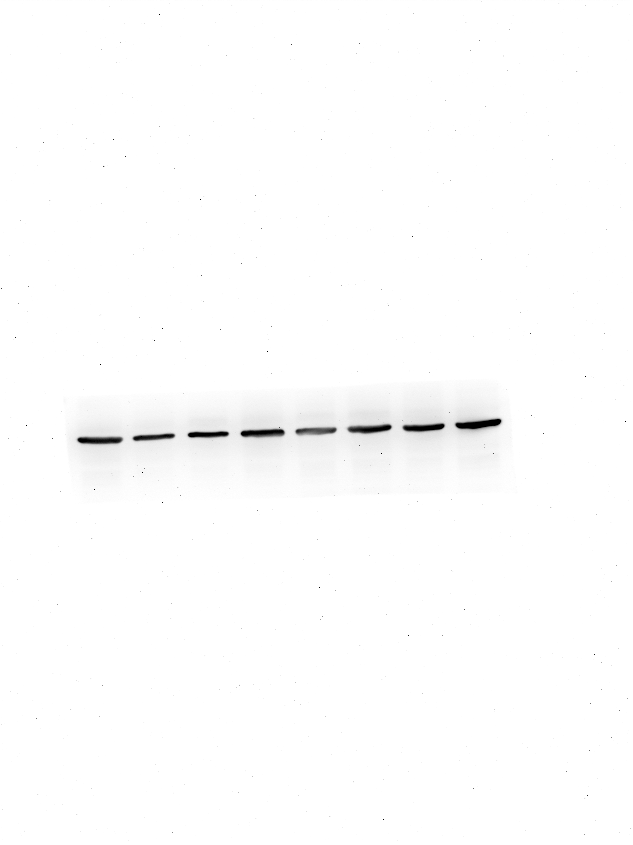


**β-actin**

**45kDa**

Supplement Figure 1C: Full unedited gel/blot for Figure 4C (GSDMD/GSDMD-N)


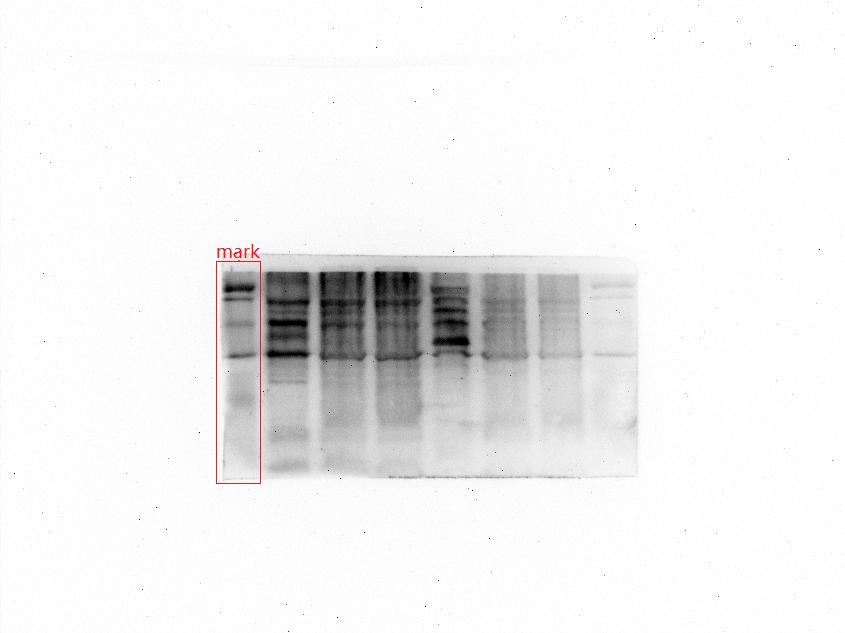


**55kDa:GSDMD GSDMD**

**40kDa**

**55kDa**

**75kDa**

**25kDa:GSDMD-N**

**25kDa**

Supplement Figure 1D: Full unedited gel/blot for Figure 4C (β-actin)


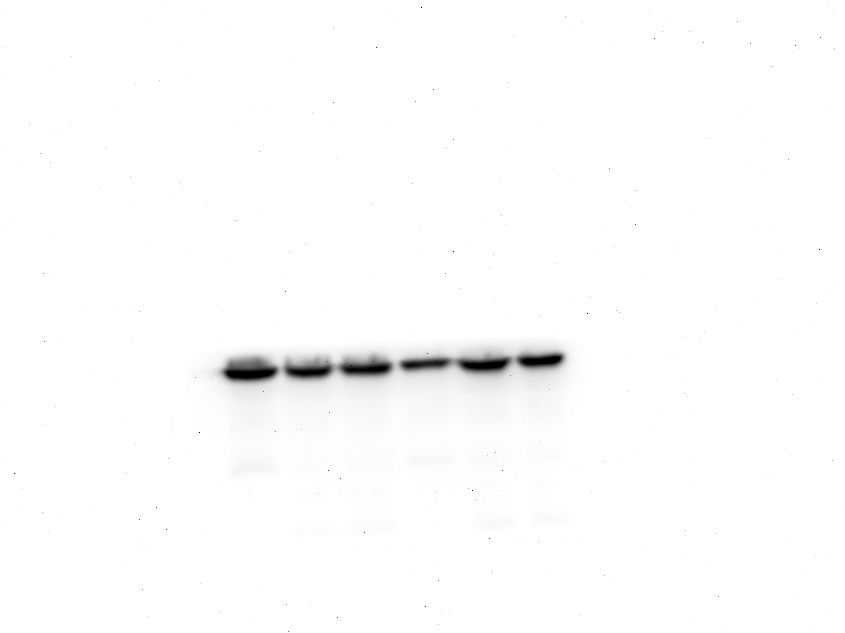


**45kDa**

**β-actin**

Supplement Figure 1E: Full unedited gel/blot for Figure 5A (NLRP3)


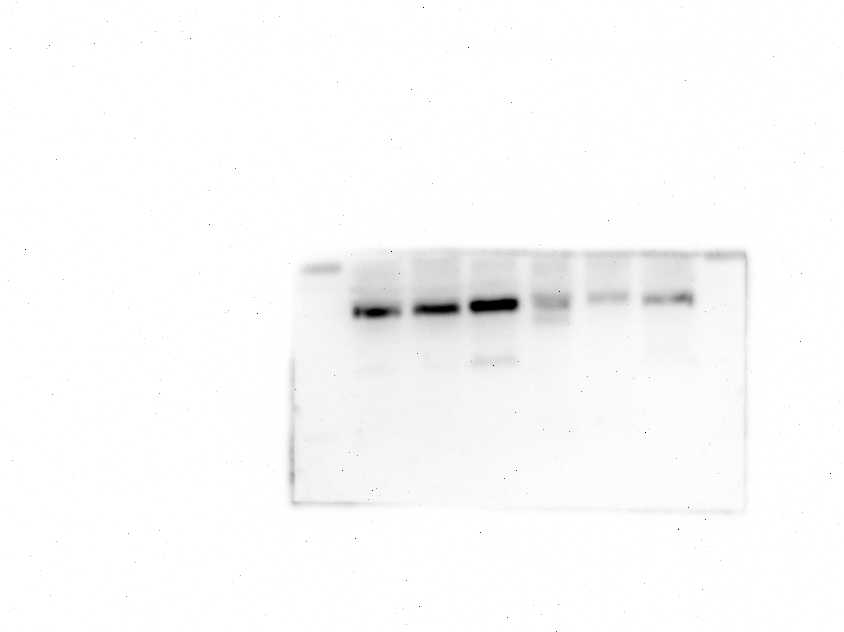


**NLRP3**

**110kDa**

Supplement Figure 1F: Full unedited gel/blot for Figure 5A (ASC)


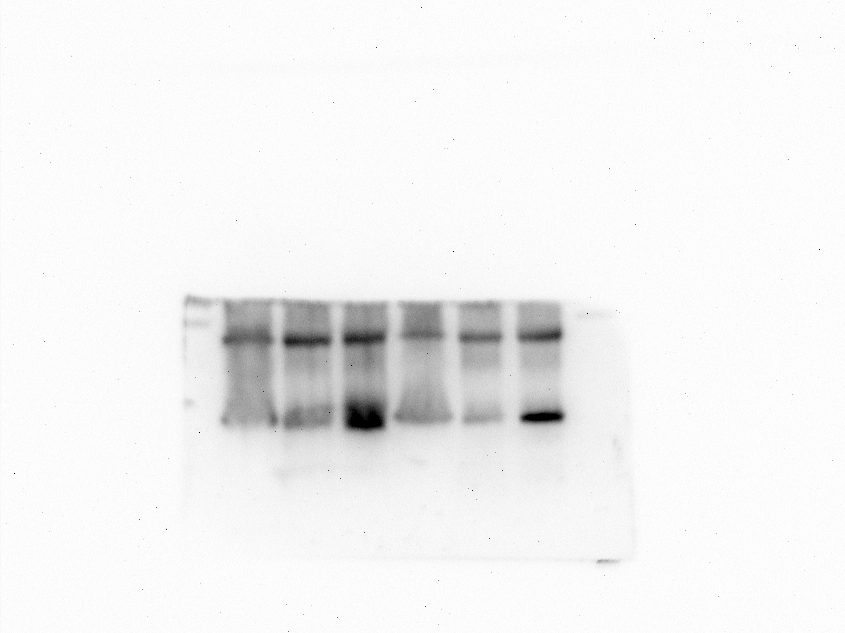


**ASC**

**22 kDa**

Supplement Figure 1G: Full unedited gel/blot for Figure 5A (pro-caspase1/cleave-caspase 1)


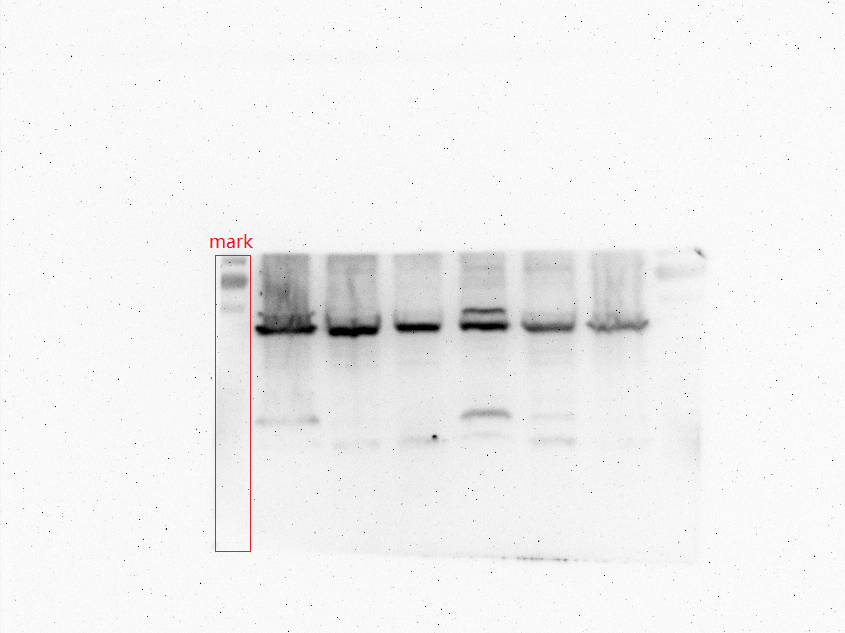


**48 kDa: pro-caspase1**

**22 kDa: cleave-caspase1**

Supplement Figure 1H: Full unedited gel/blot for Figure 5A (IL-18)


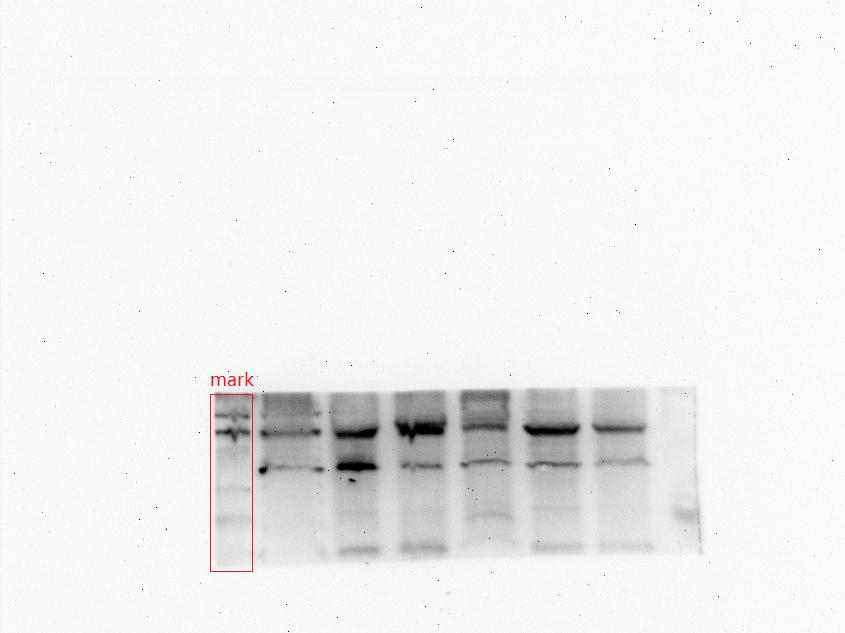


**20 kDa: IL-18**

Supplement Figure 1I: Full unedited gel/blot for Figure 5A (pro IL-1β/mature IL-1β)


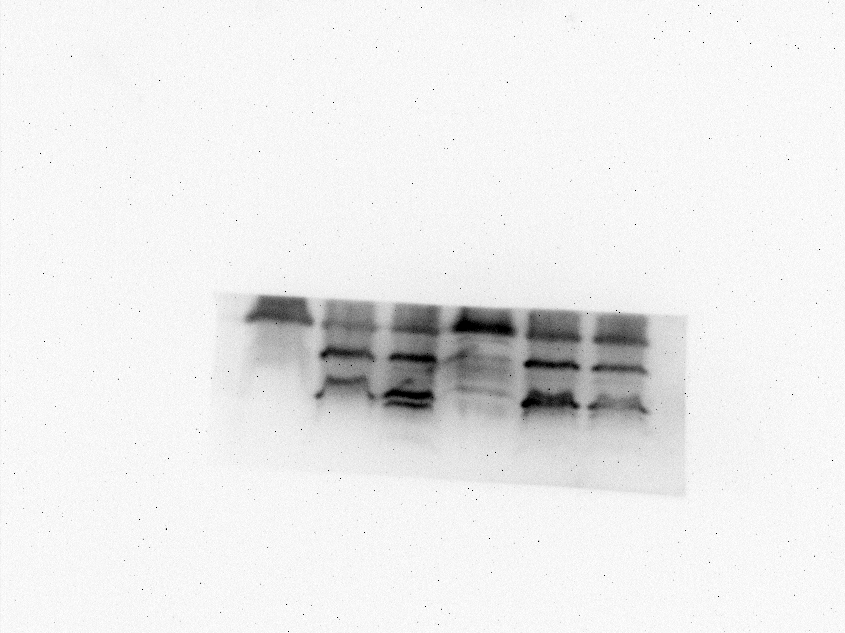


**31 kDa: pro IL-1β**

**17 kDa: mature IL-1β**

Supplement Figure 1J: Full unedited gel/blot for Figure 5A (β-actin)


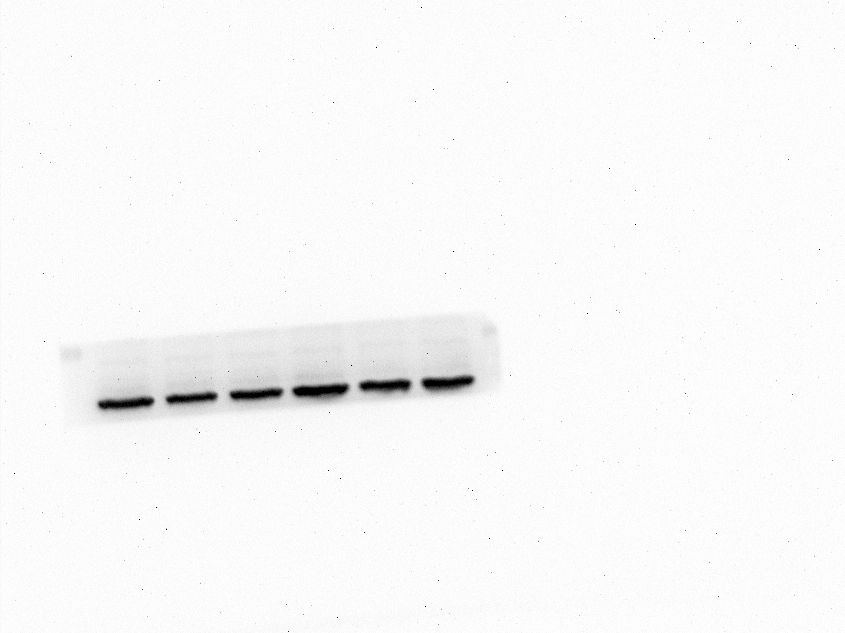


**β-actin**

**45 kDa**

Supplement Figure 1K: Full unedited gel/blot for Figure 5C (P65)


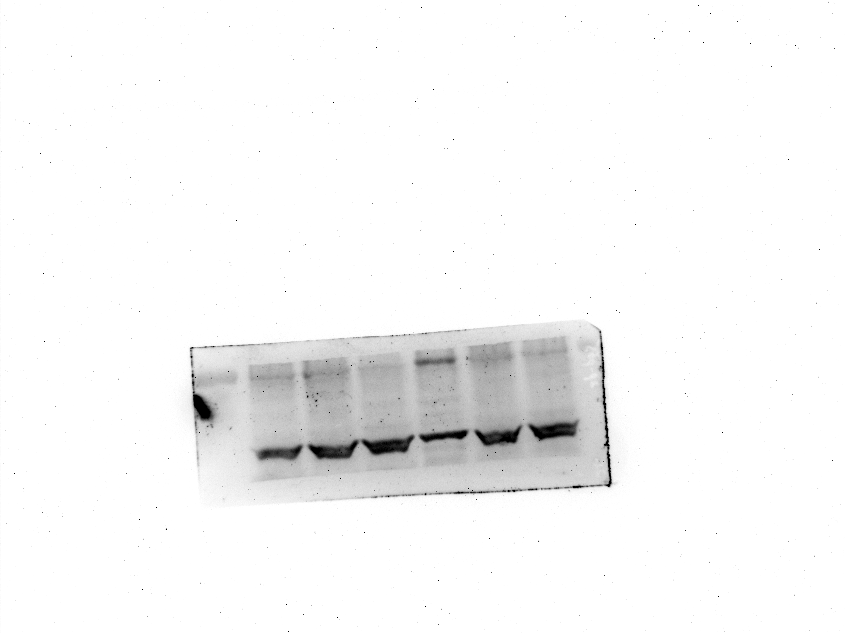


**P65**

**65 kDa**

Supplement Figure 1L: Full unedited gel/blot for Figure 5C (p-P65)


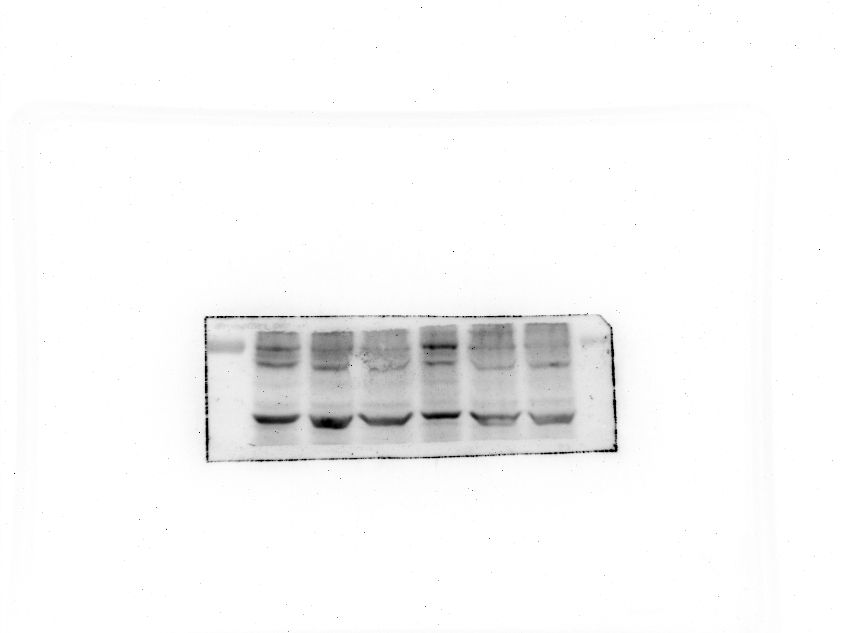


**p-P65**

**65 kDa**

Supplement Figure 1M: Full unedited gel/blot for Figure 5C (β-actin)


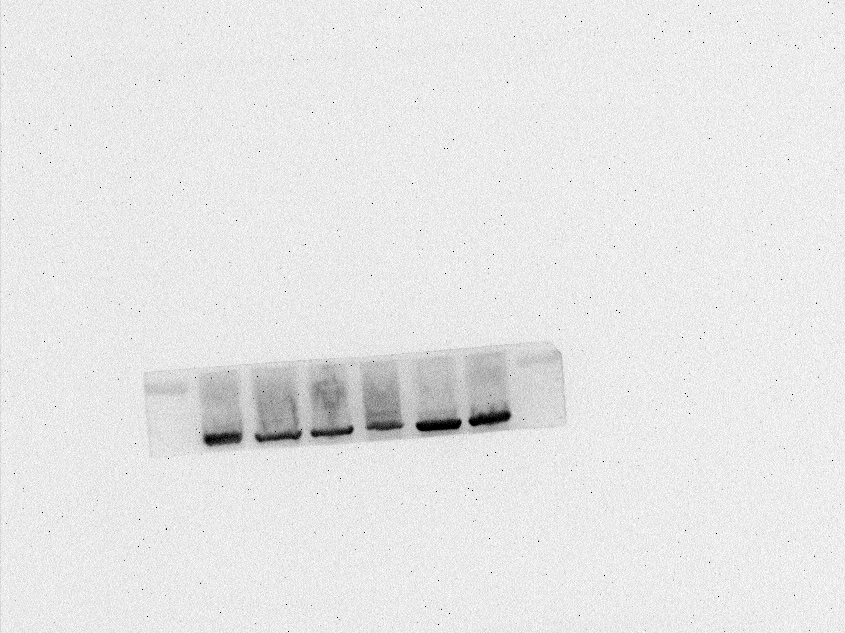


**β-actin**

**45 kDa**
