# Supplementary material for: Absence in CX3CR1 receptor signaling promotes post‐ischemic stroke cognitive function recovery through suppressed microglial pyroptosis in mice
Source: CNS Neurosci Ther. 2024 Feb 7;30(2):e14551. doi: 10.1111/cns.14551 (PMC10850801; doi:10.1111/cns.14551)

Full unedited gel/blot for Figure 4A：GSDMD

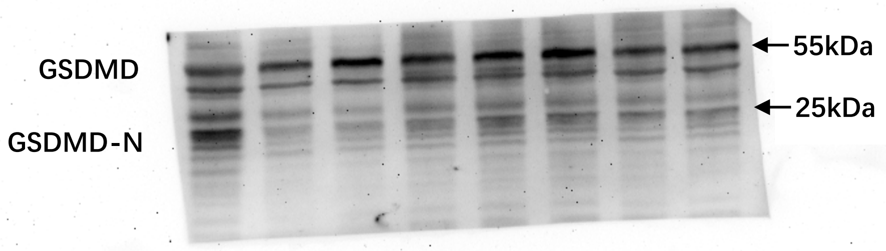

Full unedited gel/blot for Figure 4A： β-actin

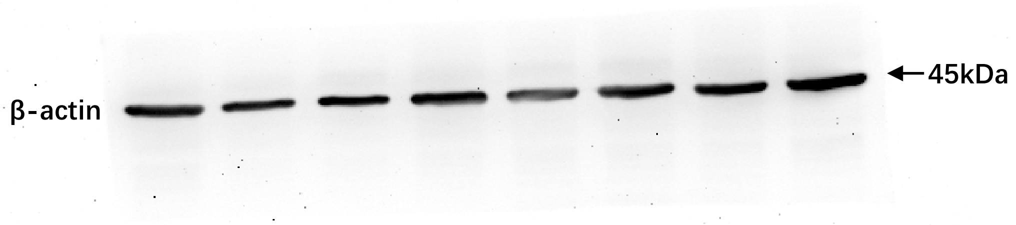

Full unedited gel/blot for Figure 4C： GSDMD

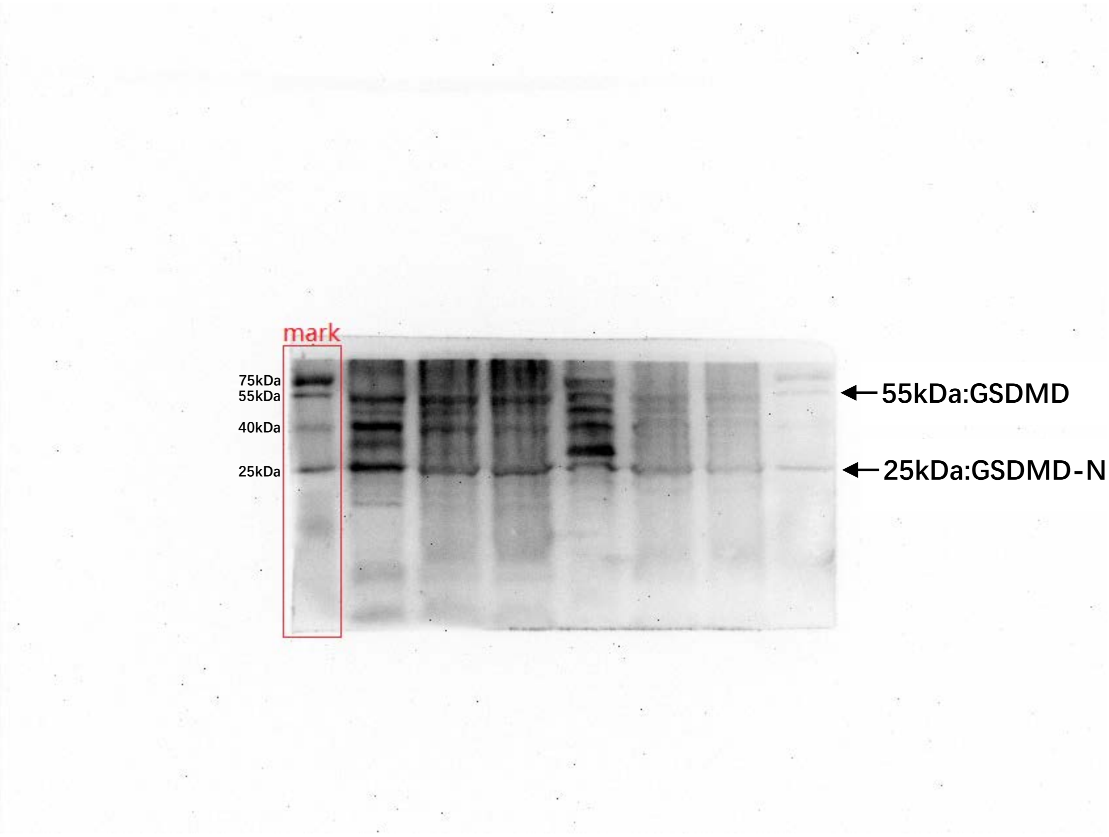

Full unedited gel/blot for Figure 4C：β-actin

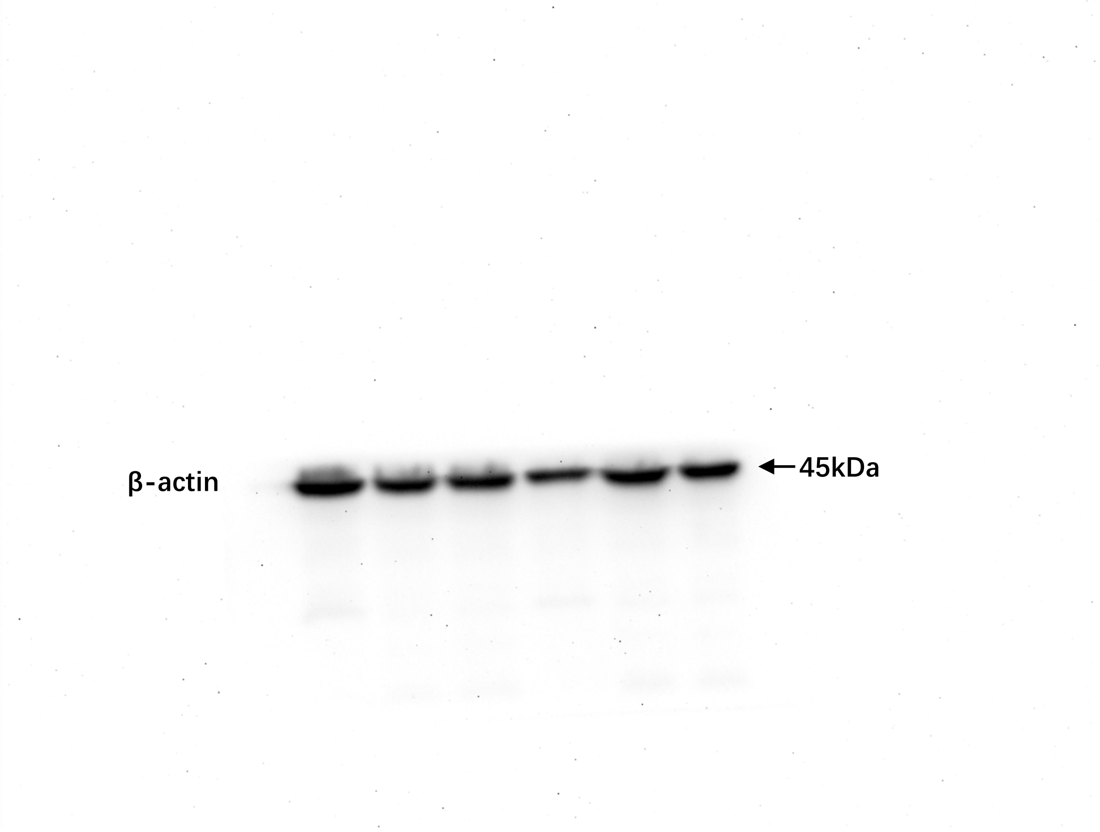

Full unedited gel/blot for Figure 5A： NLRP3

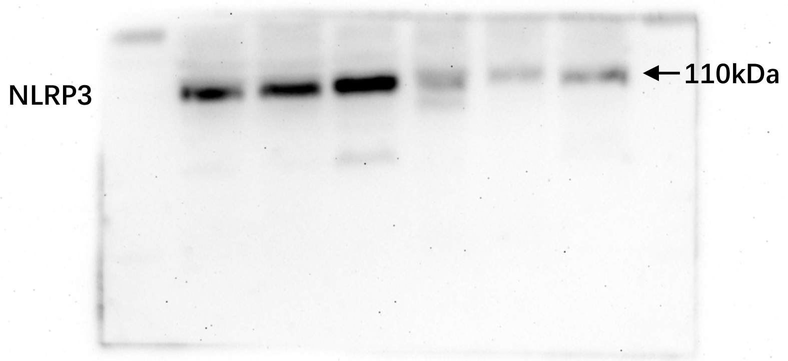

Full unedited gel/blot for Figure 5A： ASC

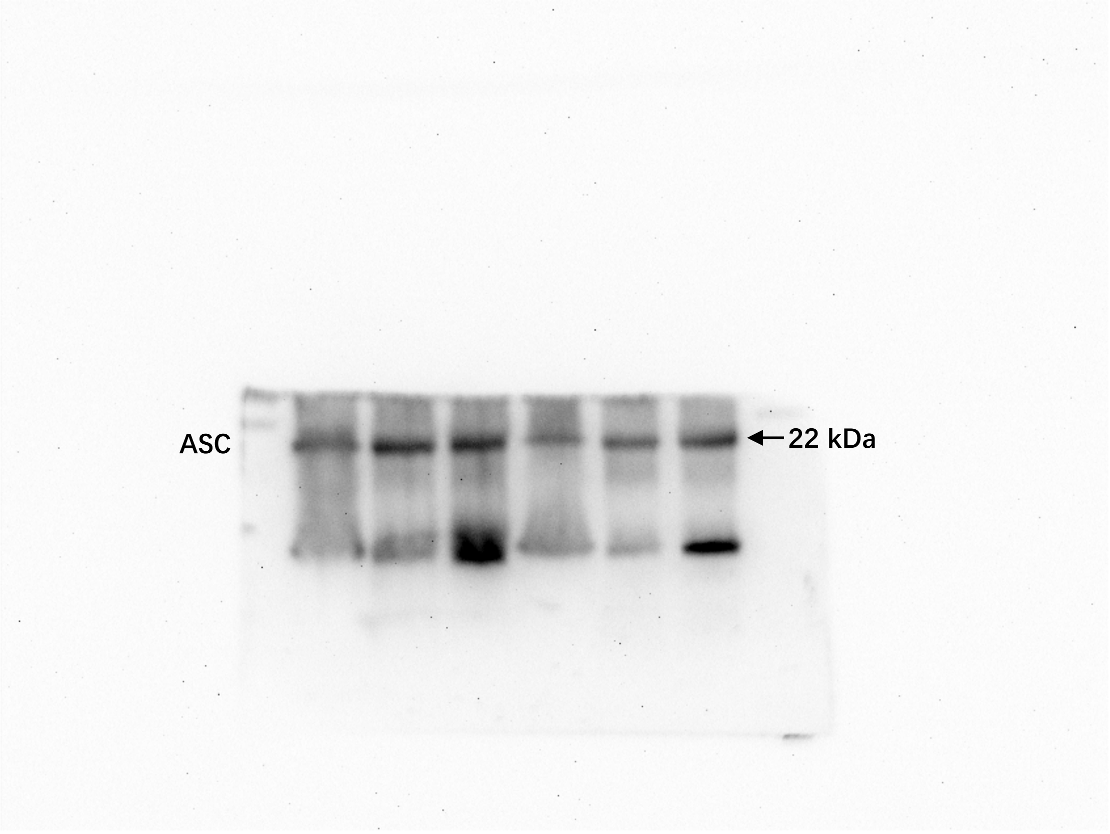

Full unedited gel/blot for Figure 5A： caspase1

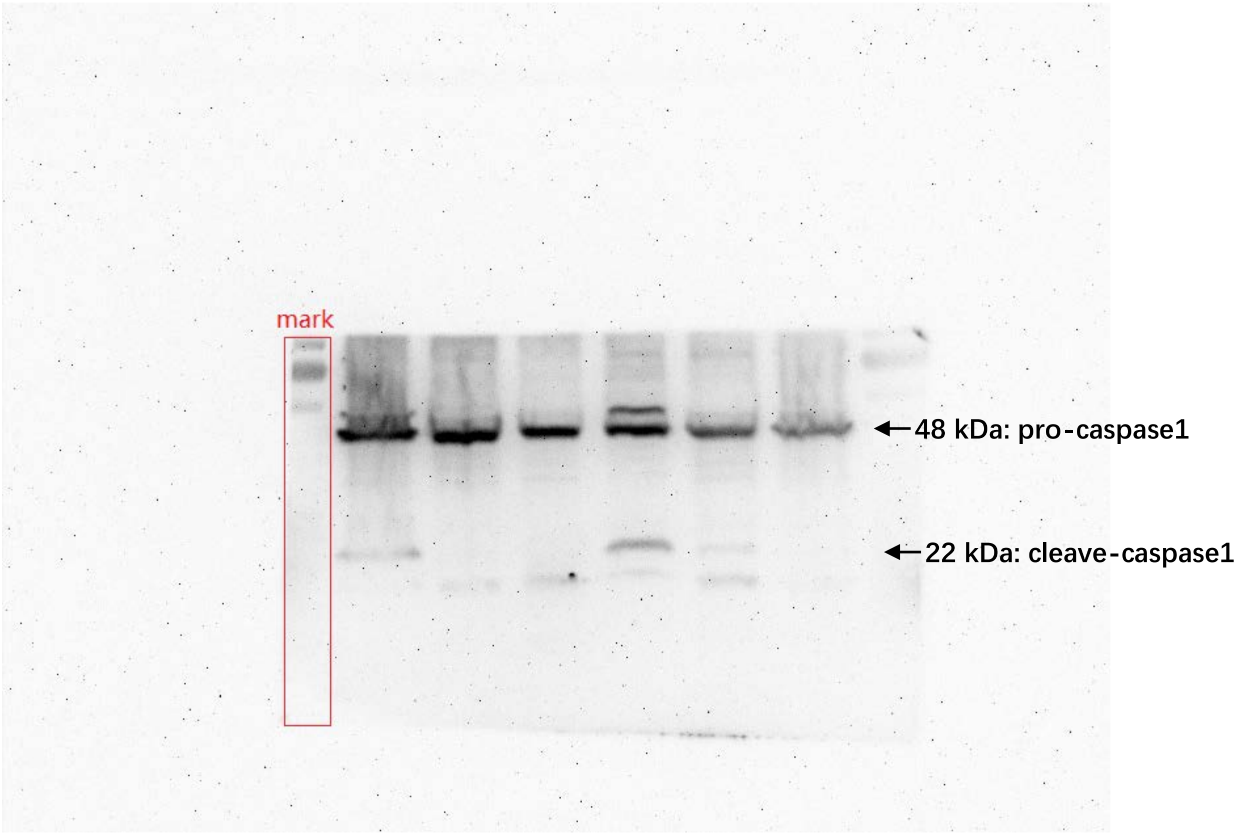

Full unedited gel/blot for Figure 5A： IL-18

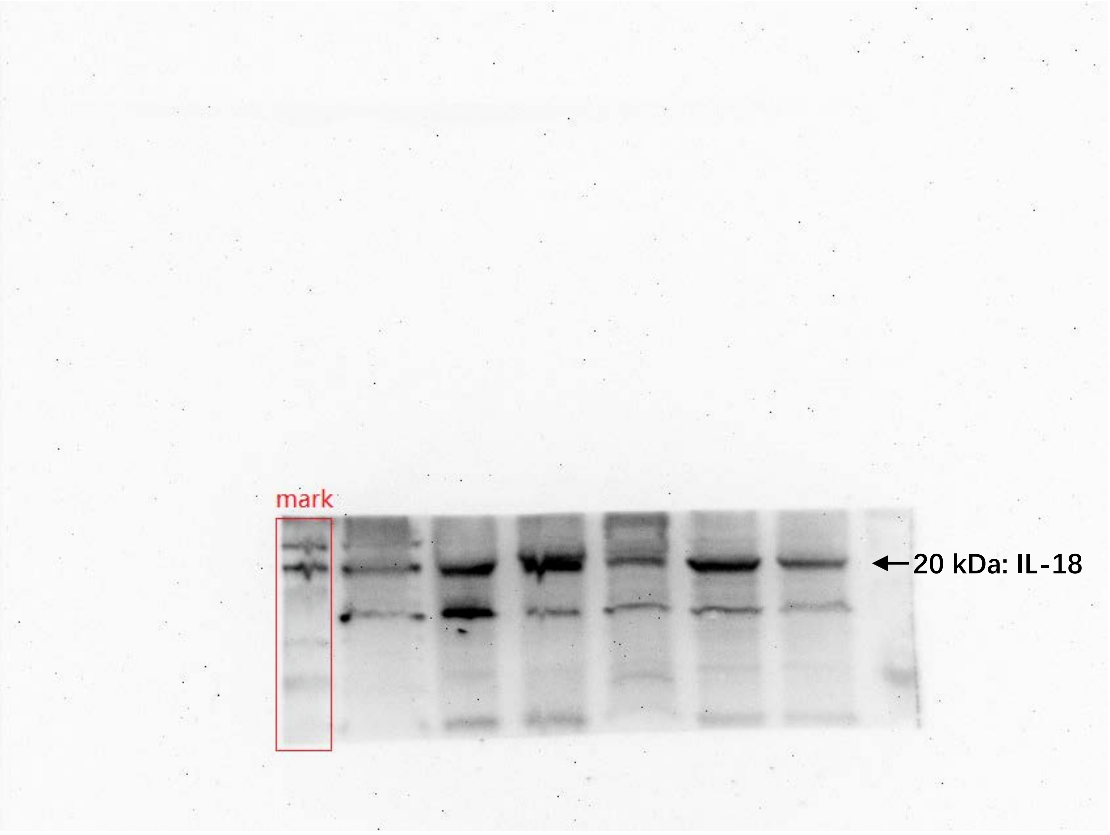

Full unedited gel/blot for Figure 5A：IL-1β

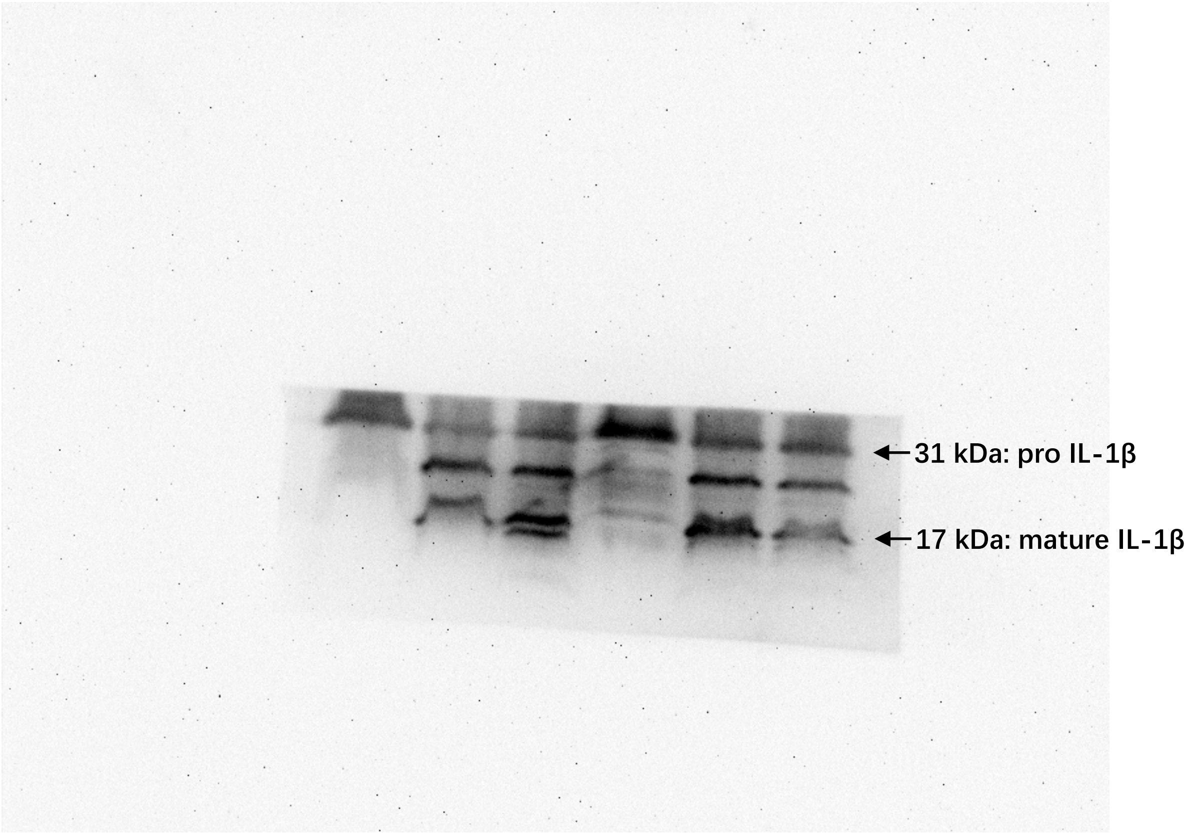

Full unedited gel/blot for Figure 5A：β-actin

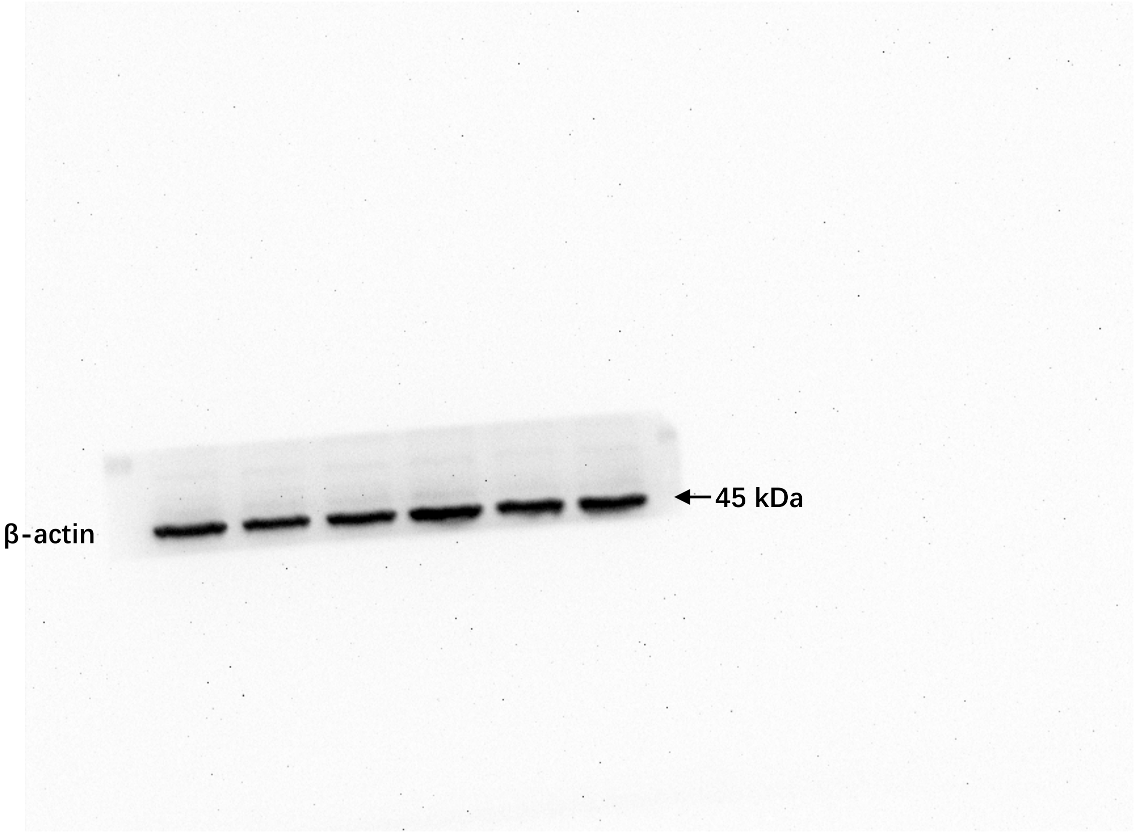

Full unedited gel/blot for Figure 5C：P65

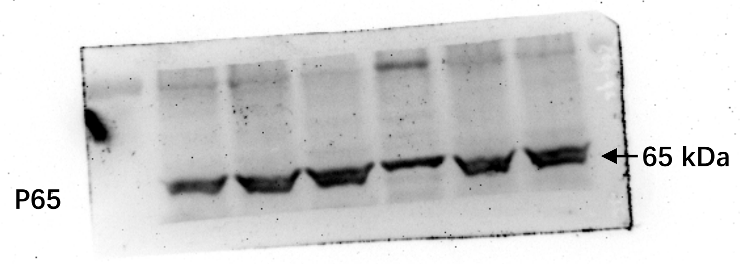

Full unedited gel/blot for Figure 5C： p-P65

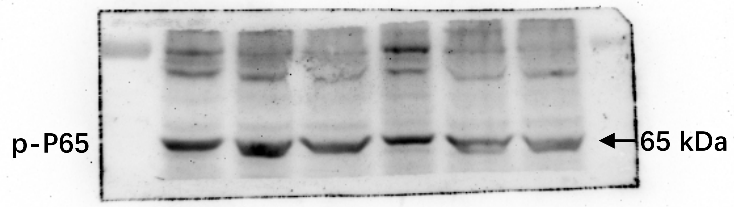

Full unedited gel/blot for Figure 5C： β-actin

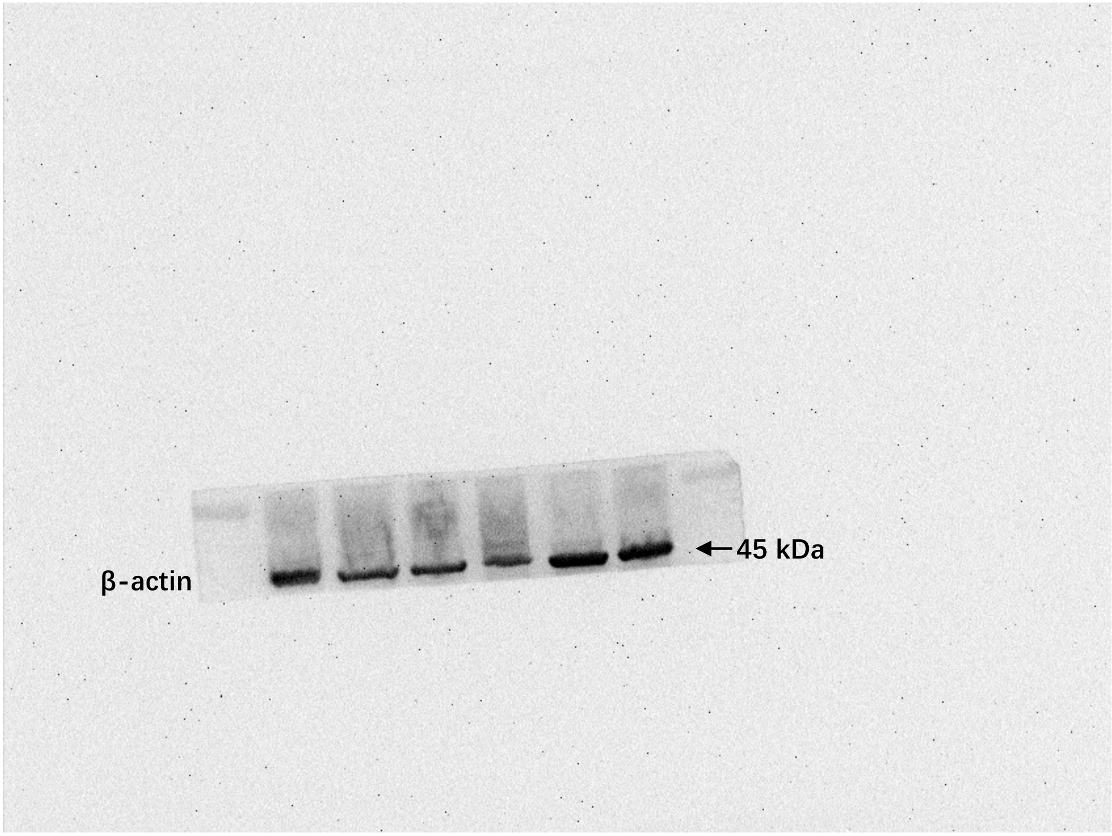

Supplement: Supplementary file 1 — Figure S1. [file CNS-30-e14551-s001.zip › Supplement Files.pdf]
